# Supplementary material for: Femtosecond concerted rotation of molecules on a 2D material interface
Source: Nat Commun. 2026 Feb 27;17:2110. doi: 10.1038/s41467-026-69801-6 (PMC12954116; doi:10.1038/s41467-026-69801-6)
Supplement: Supplementary file 1 — Supplementary information [file 41467_2026_69801_MOESM1_ESM.pdf]

Supplementary information

K. Baumgärtner[1], M. Nozaki[2], M. Reuner[3], N. Wind[4,12], M. Haniuda[2], C. Metzger[1], M. Heber[5], D. Kutnyakhov[5], F. Pressacco[5], L. Wenthaus[5], K. Hara[2], K. Chordiya[3,11], C.H. Min[6], M. Beye[5], F. Reinert[1], F. Roth[7,8], S. K. Mahatha[5,9], A. Madsen[10], T. Wehling[3,11], K. Niki[2], D. Popova-Gorelova[3,11], K. Rossnagel[6,12,13], and M. Scholz[5,10,*]

[1]Experimentelle Physik 7 and Würzburg-Dresden Cluster of Excellence ctd.qmat, Julius-Maximilians-Universität, Am Hubland, 97074 Würzburg, Germany

[2]Graduate School of Science and Engineering, Chiba Unviersity, 1-33 Yayoi-cho, Inage-ku, Chiba 263-8522, Japan

[3]I. Institute for Theoretical Physics and Centre for Free-Electron Laser Science, Universität Hamburg, Luruper Chaussee 149, 22607 Hamburg, Germany

[4]Institut für Experimentalphysik, Universität Hamburg, Luruper Chausee 149, 22761 Hamburg, Germany

[5]Deutsches Elektronen-Synchrotron DESY, Notkestrasse 85, 22607 Hamburg, Germany

[6]Institut für Experimentelle und Angewandte Physik, Christian-Albrechts-Universität zu Kiel, 24098 Kiel, Germany

[7]Institute of Experimental Physics, TU Bergakademie Freiberg, Leipziger Strasse 23, 09599 Freiberg, Germany

[8]Center for Efficient High Temperature Processes and Materials Conversion (ZeHS), Winklerstraße 5, 09599 Freiberg, Germany

[9]UGC-DAE Consortium for Scientific Research, Khandwa Road, Indore 452001, Madhya Pradesh, India

[10]European X-Ray Free-Electron Laser Facility, Holzkoppel 4, 22869 Schenefeld, Germany

[11]The Hamburg Centre for Ultrafast Imaging (CUI), Luruper Chaussee 149,

22607 Hamburg, Germany
[12]Ruprecht Haensel Laboratory, Deutsches Elektronen-Synchrotron DESY,
Notkestraße 85, 22607 Hamburg, Germany
[13]Kiel Nano, Surface and Interface Science KiNSIS, Kiel University,
Christian-Albrechts-Platz 4, 24118 Kiel, Germany
[*]To whom correspondence should be addressed; markus.scholz@desy.de.

Suppl. Note 1. Experimental analysis

1.1 LEED

The LEED image characteristic of a monolayer of CuPc molecules on $TiSe_2$ prepared at room temperature.

1.2 Valence Region Data Treatment

All valence region data was treated with a Gaussian filter for better visibility of certain features. In Fig. 2 the CuPc HOMO at t_{-1} is shown as (a) raw data and (b) processed data with an applied Gaussian filter with kernel size 5x5.

1.3 CuPc HOMO evaluation

Below the Fermi level at a binding energy of 310 meV, we identify the CuPc HOMO as an intensity modulated ring centered around the Γ-point with a radius of $(1.67 \pm 0.01)\,\text{Å}^{-1}$. Substrate derived intensities appear at the Γ-points and M-points. To disentangle molecular intensities from substrate intensities, the data was masked in momentum-energy space as shown in Fig. 3. The masked data was then separately integrated within an energy window of -(295 $\pm$ 220) meV for both the HOMO and the substrate features as shown in Fig. 3 b and c, respectively. The integration window of the CuPc HOMO in Fig. 1 d of the main manuscript is readjusted for each time step according to the shifting binding energy of the HOMO.

We observe a depletion of the HOMO with a time constant of $\tau_{HOMO,1} = (222 \pm 50)$ fs, followed by a repopulation with a time constant of $\tau_{HOMO,2} = (769 \pm 250)$ fs. Following the population dynamics, we observe a transient energetic broadening of the HOMO's FWHM by up to $\sim 12\%$ (see Fig. 4 a, black line) next to a shift towards smaller binding energies (see

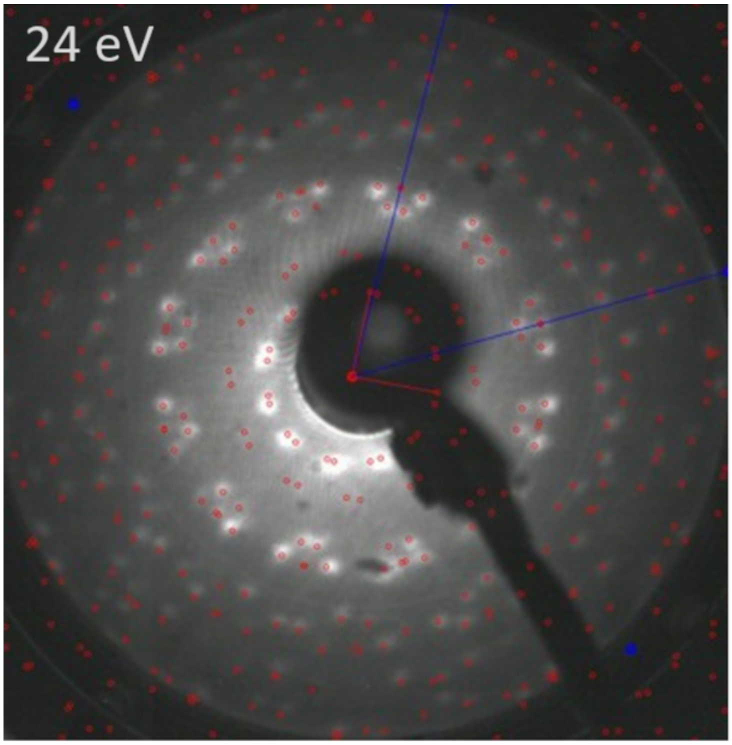

Supplementary Figure 1: LEED image of a monolayer of CuPc adsorbed on $TiSe_2$ ($E_{kin} = 24$ eV). Molecular reflections are marked with red dots and the substrate spots are marked with blue dots. The lines indicate one symmetry set of the respective reciprocal lattices.

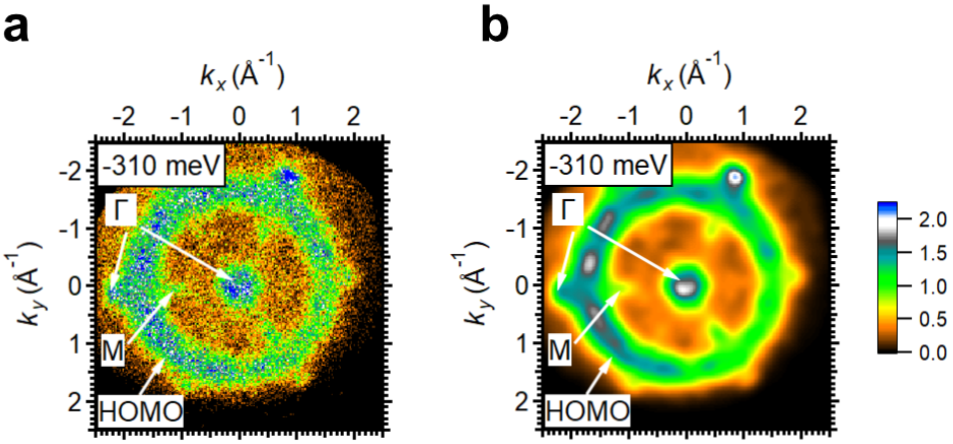

Supplementary Figure 2: CuPc HOMO at t_{-1} shown as (a) raw data and (b) processed data with a Gaussian filter with kernel size 5x5.

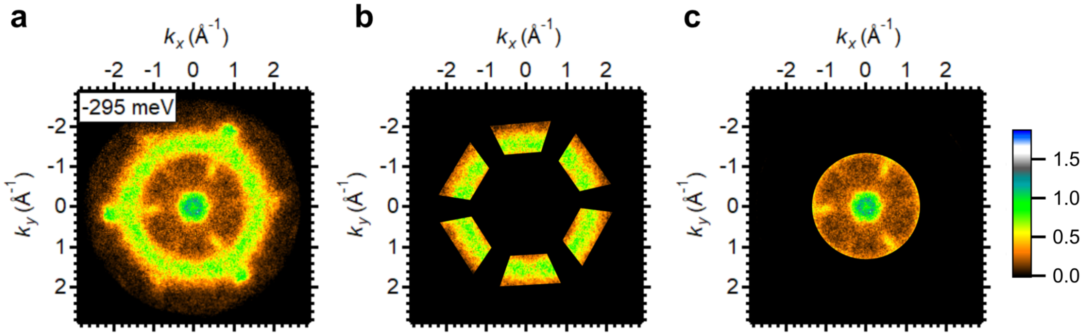

Supplementary Figure 3: In (a) the experimental momentum distribution of a monolayer CuPc/$TiSe_2$ at the HOMO energy is shown. In (b) the integrated areas masked for the HOMO evaluation and in (c) for the substrate are shown.

Fig. 4 a, red line). Such an energetic broadening was previously observed for molecular orbitals involved in charge transfer (*1*). The transient HOMO ring width and radius in momentum space,

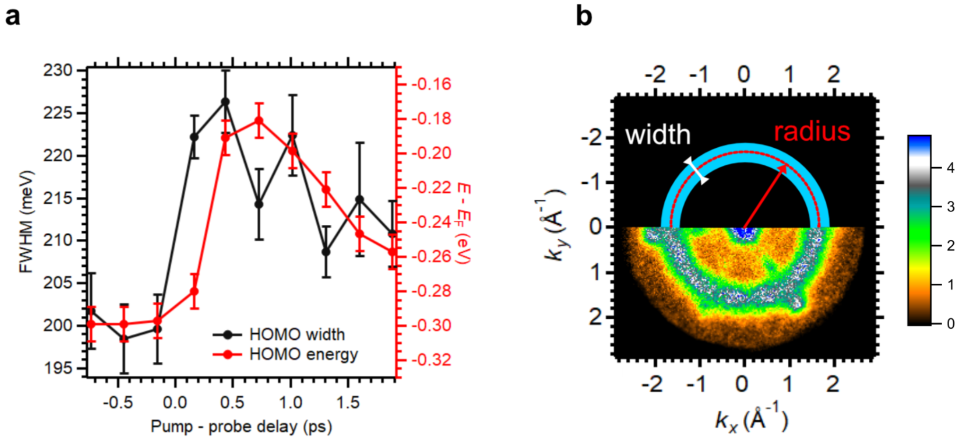

Supplementary Figure 4: (a) Transient energetic width (FWHM) of the CuPc HOMO (black, left axis) and transient energetic position of the HOMO (red, left axis). 1σ error-bars are shown. (b) Sketch of the evaluated transient properties 'HOMO ring radius' and 'HOMO ring width in momentum space' as shown in Fig. 3 e of the main manuscript.

as shown in Fig. 3 e, are evaluated according to the sketch in Fig. 4 b.

State	τ_1 (fs)	τ_2 (fs)
CuPc LUMO	94 ± 8 (decrease)	-
Ti 3*d*	243 ± 50 (increase)	503 ± 250 (decrease)
CuPc HOMO	222 ± 50 (decrease)	769 ± 250 (increase)
C 1*s* peak 1 ($CuPc^0$)	264 ± 72 (decrease)	588 ± 50 (increase)
C 1*s* peak 4 ($CuPc^+$)	403 ± 150 (increase)	500 ± 150 (decrease)

Supplementary Table 1: Time constants evaluated for the transient intensities of CuPc and $TiSe_2$ states shown in Fig. 3 b in the main text.

1.4 Time-constants evaluated for the transient intensities of CuPc/$TiSe_2$ valence and core-level states

Tab. 1 lists the evaluated time constants for the depletion and population of states presented in Fig. 3 b in the main text.

1.5 LAPE considerations

The sample was excited with 1.55 eV *p*-polarized pump pulses. If laser-assisted photoemission (LAPE) were present, replicas of prominent spectral features would be expected. In particular, a sideband of the Fermi edge should appear 1.55 eV above EF, and the strong CuPc HOMO signal at –310 meV would produce a LAPE replica near 1.24 eV above EF. Neither feature is observed.

Furthermore, the momentum distribution of the transient population is inconsistent with the symmetry expected for a LAPE replica. At 180 meV above EF, the measured photoemission pattern exhibits the threefold symmetry of the Ti 3*d* conduction band, not the momentum distribution of the Se 4*p* valence states at –1.37 eV.

The extracted decay time of the transient molecular LUMO population (94 fs) is comparable to the instrument response function, providing an instrument-limited upper bound for the true decay. This behavior differs from the quasi-instantaneous onset expected for LAPE sidebands.

Taken together, the absence of replica features, the distinct *k*-space symmetry, and the observed fluence and polarization behavior demonstrate that the transient signal reflects genuine population dynamics and is not a LAPE artifact.

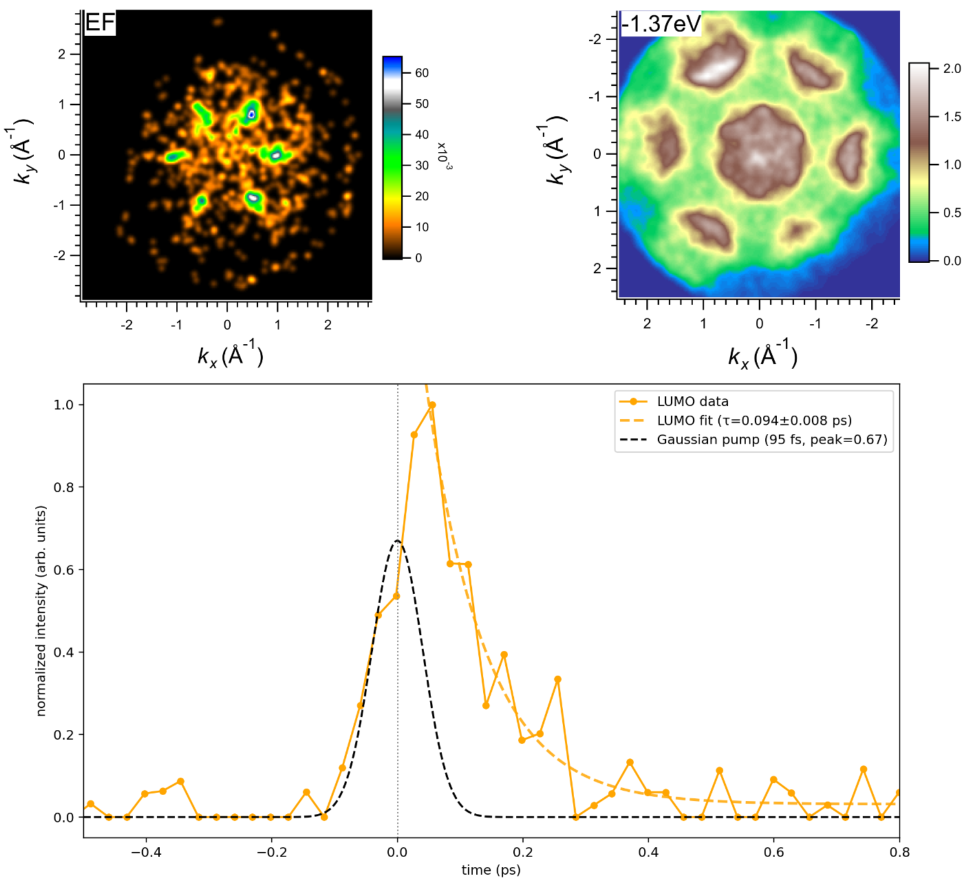

Supplementary Figure 5: Upper part) Iso-energy cut with at time zero with an energy integration window of 200 meV around the energy given in the respective picture. Lower part) Intensity population of LUMO with τ = 94 fs and Gaussian pump pulse with a FWHM of 95 fs.

1.6 C 1*s* momentum map evaluation

The XPD data in Fig. 4 b and c of the main manuscript show the C 1*s* signal of CuPc/$TiSe_2$ after subtraction of the inelastic background. To extract the molecular geometries at t_{-1} and at t_1, the experimental momentum maps are compared to simulated momentum maps of 42 different sample geometries. Their quantitative agreement is evaluated by means of a R-factor analysis according to the procedure shown in (*2*). The 42 evaluated R-factors are shown in Fig. 6. R factor values are shown in (a), details on the studied models are given in (b) and (d), side views of the studied models are shown in (c). The models corresponding to the smallest R-factor are viewed as the closest fits to the experiment (shown in Fig. 4 d of the main text).

a

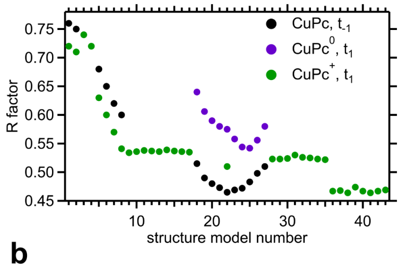

b

#	shape	in-plane rotation	#	shape	in-plane rotation
1	2	0°	23	1	-5°
2	4	0°	24	1	-10°
3	4,+0.5 Å	0°	25	1	-15°
4	4, -0.5 Å	0°	26	1	-20°
5	6	0°	27	1	-25°
6	5	0°	28	8	0°
7	7	0°	29	8	+5°
8	3	+25°	30	8	+15°
9	3	+20°	31	8	+25°
10	3	+15°	32	8	+35°
11	3	+10°	33	8	+45°
12	3	+5°	34	8	+55°
13	3	0°	35	8	+65°
14	3	-5°	36	9	0°
15	3	-10°	37	9	+5°
16	3	-15°	38	9	+15°
17	3	-20°	39	9	+25°
18	1	+20°	40	9	+35°
19	1	+15°	41	9	+45°
20	1	+10°	42	9	+55°
21	1	+5°	43	9	+65°
22	1	0°			

c

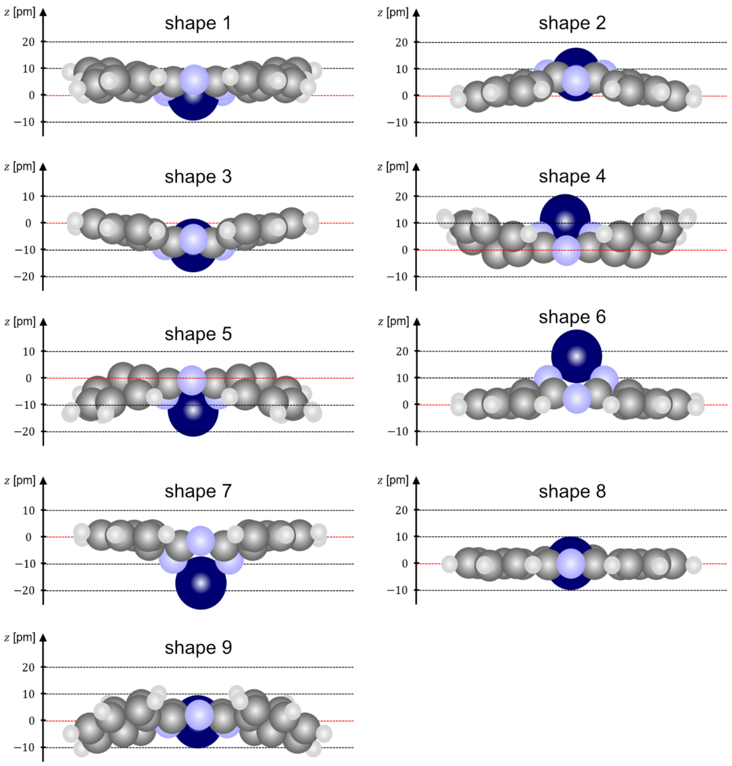

d

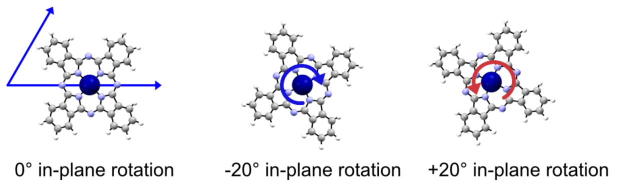

´

Supplementary Figure 6: R-factor analysis of CuPc molecules. (a) R-Factor for different structural models. (b) structural models for different in-plane rotations. (c) Illustration of structural models. Note that the atomic radii are scaled by a factor of 1/7 for better visibility. (d) Sketch of in-plane orientations.

1.7 C 1*s* Fluence-dependent dynamics

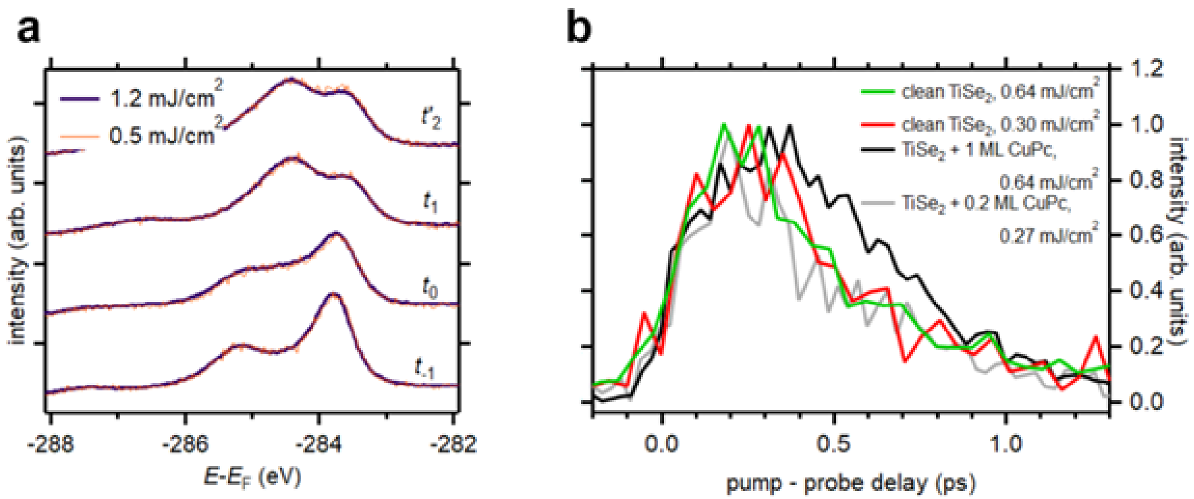

Supplementary Figure 7: Fluence-dependent dynamics. (a) C 1*s* core-level evolution of $TiSe_2$ covered by a monolayer of CuPc for different pump laser fluences. (b) Population dynamics of the Ti 3*d* conduction band for varying pump laser fluences for pristine $TiSe_2$, $TiSe_2$ covered by 0.2 monolayers of CuPc, and $TiSe_2$ covered by a monolayer of CuPc.

Fig. 7 a shows the CuPc C 1*s* core-level spectrum for different pump fluences. Since the spectral shape of the C 1*s* core emission does not change when the excitation fluence is nearly doubled from 0.5 mJ/cm^2 to 1.2 mJ/cm^2, we conclude that the observed dynamics are not fluence dependent within the studied region. In the Ti 3*d* conduction band, efficient hot hole transfer into the CuPc adsorbates is represented by a prolonged population increase after t_0. To evaluate whether the efficient hot hole transfer depends on the pump laser fluence or on the adsorbate coverage, three samples with different adsorbate coverages are studied for different excitation fluences. Doubling the excitation fluence on pristine $TiSe_2$ does not affect the Ti 3*d* population dynamics. $TiSe_2$ covered by $\sim$0.2 monolayers of CuPc also follows the same dynamics as pristine $TiSe_2$. Only for the $TiSe_2$ sample covered by a monolayer of CuPc, a prolonged increase of the Ti 3*d* population is observed.

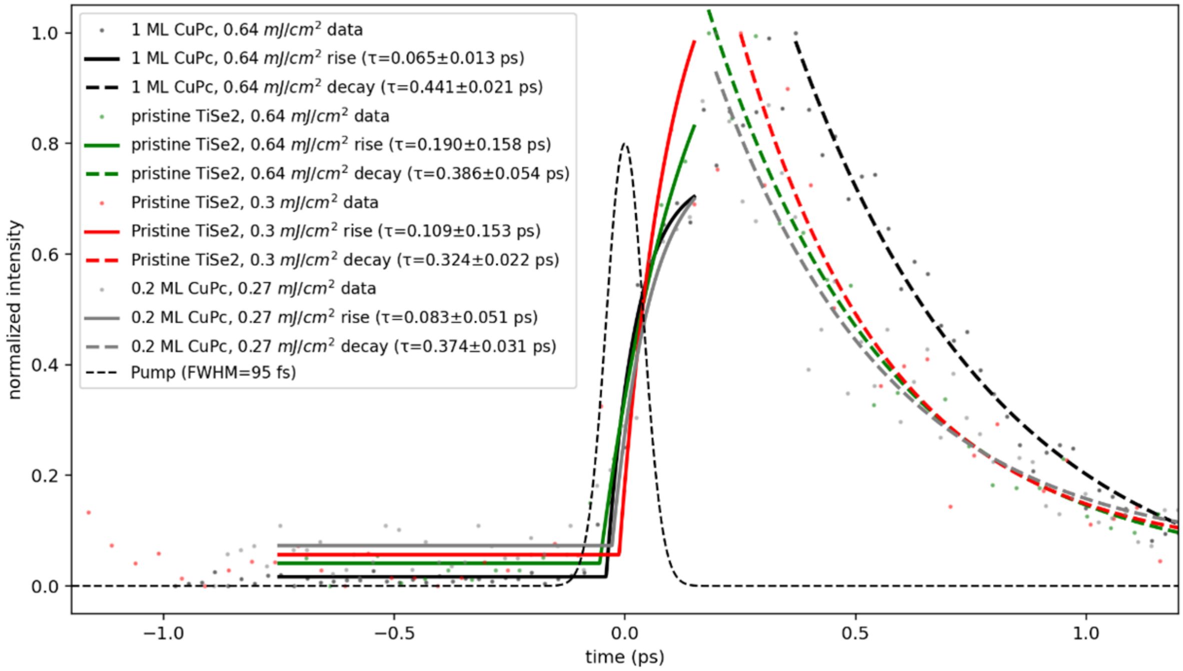

Supplementary Figure 8: Population dynamics of pristine $TiSe_2$ and CuPc/$TiSe_2$ samples as well as fitted rising and decay functions. In addition, a Gaussian peak with a FWHM of 95 fs at t_0=0 is shown.

	fluence	τ_{rising}	τ_{decay}	t_{max}
$TiSe_2$ + 1 ML CuPc	0.64 mJ/cm^2	(0.065 ± 0.013) ps	(0.443 ± 0.021) ps	(0.369 ± 0.041) ps
Pristine $TiSe_2$	0.64 mJ/cm^2	(0.190 ± 0.158) ps	(0.386 ± 0.054) ps	(0.181 ± 0.054) ps
Pristine $TiSe_2$	0.30 mJ/cm^2	(0.109 ± 0.153) ps	(0.340 ± 0.022) ps	(0.251 ± 0.013) ps
$TiSe_2$ + 0.2 ML CuPc	0.27 mJ/cm^2	(0.083 ± 0.051) ps	(0.393 ± 0.031) ps	(0.197 ± 0.028) ps

Supplementary Table 2: Extracted single exponential rising and decay constants as well as maximum of intensity for pristine $TiSe_2$ and CuPc/$TiSe_2$ samples.

Suppl. Note 2. Theoretical modelling

2.1 Dielectric model of the photoexcited system

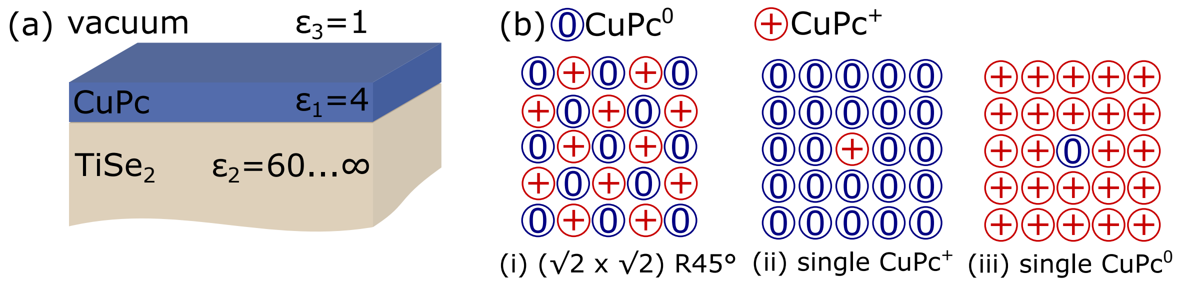

´

Supplementary Figure 9: Dielectric model of monolayer CuPc on $TiSe_2$. (a) Side view. (b) Top view. For the photoexcited state three different arrangements (i-iii) of neutral ($CuPc^0$) and positively charged molecules ($CuPc^+$) are considered.

For a qualitative understanding of the excitation induced shifts in the core levels of CuPc and the CuPc HOMO with respect to the Se $4p$ states $TiSe_2$ we consider a dielectric model of the CuPc on $TiSe_2$ system.

We assume that after photoexcitation a hole is transferred from $TiSe_2$ to a certain fraction of CuPc molecules. Hence, this fraction of CuPc molecules will be positively charged (+) while the rest of the molecules will remain neutral. The $TiSe_2$ carries the opposite negative charge (−). The resultant electrostatic potential $U(\mathbf{r})$ is calculated using the model sketched in Fig. 9: the CuPc molecules are assumed to form a square lattice with lattice constant $a = 14.5$Å above the $TiSe_2$ substrate (*3*). To describe the dielectric screening in this setup, the CuPc molecules are represented as dielectric layer $\varepsilon_1 = 4$, c.f. Ref. (*4*), of thickness $h = 3.95$Å. In our relaxed structure the vertical distance between the upmost Se (Ti) atom and the CuPc molecule is 3.1 Å (4.8 Å). We work with an effective dielectric height h = 3.95 Å of CuPc, which is the average between the CuPc to Se and CuPc to Ti distance. The $TiSe_2$ substrate is modeled as half-space with dielectric constant ε_2, while we assume vacuum above the CuPc layer($\varepsilon_3 = 1$). For the electron doped $TiSe_2$ we consider $\varepsilon_2 = 60$ (c.f. Ref. (*5*)) and, most simplistically, a perfect metal $\varepsilon_2 = \infty$.

Since this pattern of positively charged and neutral CuPc molecules after photoexcitation is unknown and also inhomogeneities are likely, we consider three cases here: (i) a $(\sqrt(2) \times \sqrt(2))$

R45° superstructure with alternating positively charged and neutral CuPc, (ii) 10×10 supercell with a single positively charged CuPc surrounded by otherwise neutral molecules, and (iii) 10×10 supercell where all but one CuPc molecules are positively charged. In all cases, the positively charged CuPc molecules are modeled as Gaussian surface charge density $\rho(\mathbf{r}) = \frac{1}{2\pi d^2}\exp(-\frac{\mathbf{r}^2}{2d^2})$ of width $d = 5$Å leading to a total charge of $+1e$.

The average vertical electrostatic potential energy difference between the $TiSe_2$ surface and the CuPc layer reads $U_\perp \approx \frac{4\pi\bar{\sigma}}{\varepsilon_1}h$, where $\bar{\sigma}$ is the laterally averaged charge density of $+1e$ per supercell.

Following Refs. (*6, 7*), we obtain the Fourier space representation of the potential modulations inside the CuPc layer $U_\parallel(\mathbf{q}) = \frac{2\pi e^2}{q} \cdot \frac{\rho(\mathbf{q})}{\varepsilon_{\text{eff}}^{2D}(q)}$, where $\rho(\mathbf{q})$ is the charge density at wave vector q. Following Eq. (11) from Ref. (*6*), the dielectric screening is accounted for via the effective dielectric function

$$\varepsilon_{\text{eff}}^{\text{2D}}(q) = \frac{\varepsilon_1\left[1 - \tilde{\varepsilon}_2\tilde{\varepsilon}_3 e^{-2qh}\right]}{1 + \left[\tilde{\varepsilon}_2 + \tilde{\varepsilon}_3\right]e^{-qh} + \tilde{\varepsilon}_2\tilde{\varepsilon}_3 e^{-2qh}} \tag{1}$$

with $\tilde{\varepsilon}_j = \frac{\varepsilon_1 - \varepsilon_j}{\varepsilon_1 + \varepsilon_j}$ and $\varepsilon_{1,2,3}$ being the dielectric constants of CuPc, $TiSe_2$ and the vacuum above the sample respectively. The Fourier transformation from reciprocal to real space then yields the potential energy modulations $U_\parallel(r)$ inside the CuPc layer. Defining the average potential energy at the $TiSe_2$ surface as zero energy, yields the potential at different lateral positions inside the CuPc layer relative to $TiSe_2$, $U(r) = U_\parallel(r) + U_\perp$ shown in Fig. 10.

The models considering for the $TiSe_2$ surface $\varepsilon_2 = 60$ and $\varepsilon_2 = \infty$ turn out to yield almost identical potential energy modulations inside the CuPc layer. The shift of the CuPc potential energies relative to the $TiSe_2$ levels varies with the total transferred charge. Independently, of the charge pattern (i, ii, or iii), the potential energy *difference* between positively charged and neutral CuPc centers is always ≈ 0.3 eV.

A comparison of potential energy at the CuPc centers relative to the $TiSe_2$ surface to experimentally observed shifts in the CuPc core levels and valence orbital energies relative to the $TiSe_2$ valence orbitals upon photoexcitation is given in Tab. 3.

While there are clearly quantitative uncertainties in the theoretical model at hand, the model qualitatively captures the excitation induced relative level shifts seen in the experiment. Indeed, we note that we did not optimize the model parameters for a best fit of the experiments but rather took all parameter estimates from the literature or our experiments. Thus, the qualitative match between theory and experiment indicates that the observed shifts in spectral lines upon photoexcitation are to a significant extent of electrostatic nature.

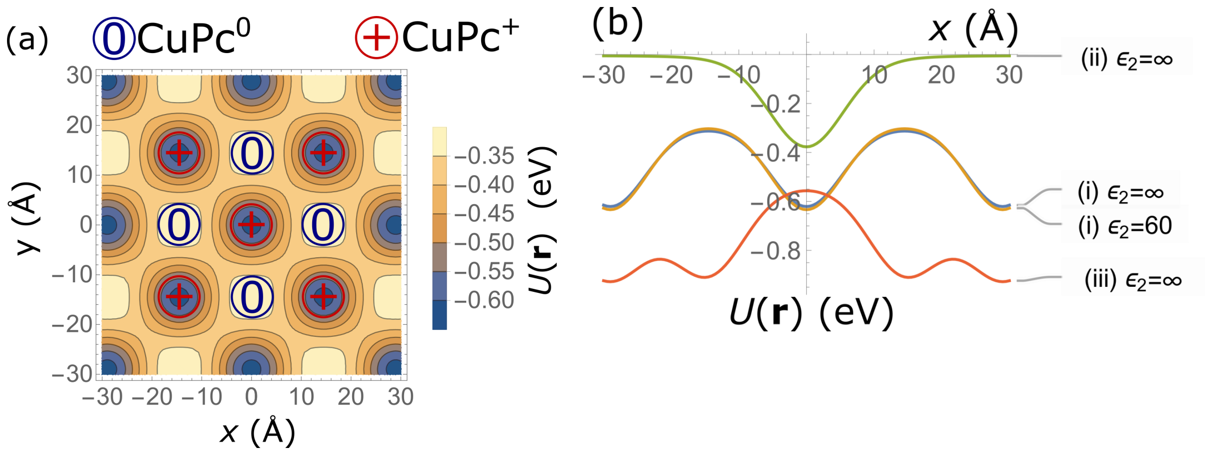

Supplementary Figure 10: Electrostatic potential $U(r)$ inside the CuPc layer relative to the $TiSe_2$ surface. (a) Electrostatic potential energy in the CuPc plane assuming a $(\sqrt(2) \times \sqrt(2))$ R45° superstructure with alternating positively charged and neutral CuPc, i.e. structure (i). (b) Potential energy along the x-direction for structure (i) assuming $\varepsilon_2 = 60$ and $\varepsilon_2 = \infty$, as well as charge distribution patterns (ii) and (iii).

Model	$CuPc^+$ (eV)	$CuPc^0$ (eV)
(i) $(\sqrt{2} \times \sqrt{2})$ R45° cell, $\varepsilon_2 = 60$	-0.6	-0.3
(i) $(\sqrt{2} \times \sqrt{2})$ R45° cell, $\varepsilon_2 = \infty$	-0.6	-0.3
(ii) (10×10) cell, single $CuPc^+$, $\varepsilon_2 = \infty$	-0.4	0.0
(iii)(10×10) cell, single $CuPc^0$, $\varepsilon_2 = \infty$	-0.9	-0.6
Experiment	-0.843 (core level)	-0.08 (HOMO)<br>+0.05 (core level)

Supplementary Table 3: Excitation induced shifts of CuPc levels relative to $TiSe_2$: theoretical models (i-iii) versus experiment. $CuPc^+$ and $CuPc^0$ refer to positively charge and neutral molecular centers, respectively. The average potential at the $TiSe_2$ surface is taken as reference energy in the theoretical model. In the experimental data the shift in the Se $4p$ valence band peak is subtracted from the excitation induced shifts in the CuPc energy levels.

2.2 Calculations of the valence and core level momentum distribution for CuPc/$TiSe_2$

In order to study the photoelectron momentum maps (PMMs) of the highest occupied molecular orbital (HOMO) for the CuPc/$TiSe_2$ system, we performed DFT calculations using the Vienna Ab Initio Software Package (VASP) version 5.4.4 (*8*), (*9*). Based on the wave functions obtained from the DFT calculations, the PMM of the CuPc HOMO and the Molecular Orbital Projected

Density of States (MOPDOS) were simulated using kMap.py and MOPDOS@VASP, respectively (*10*). The exchange correlation effects were described by the spin-polarized generalized gradient approximation (GGA) within the Perdew-Burke-Ernzerhof (PBE) formalism (*11*). We used a plane-wave basis set with the projector augmented wave (PAW) approach with an energy cutoff of 500 eV. The Brillouin zone integration was performed on a Monkhorst-Pack of 6x6x1 grid of k-points. To account for Fermi surface broadening, a first-order Methfessel-Paxton smearing of 0.01 eV was applied (*12*). Coulomb and exchange interactions of the localized d-orbitals in the transition metal elements were treated in the framework of the Dudarev DFT+U method with an effective Hubbard parameter $U_{\mathrm{eff}} = 5.5$ eV for Ti and $U_{\mathrm{eff}} = 4.0$ eV for Cu. Thereby, U_{eff} of Ti was adjusted such that the band structure obtained for a $TiSe_2$ supercell of $1 \times 1 \times 1$ (a = b =3.53 Å, c = 3.08 Å, $\alpha = \beta = 90°$ and $\gamma = 120°$, introducing a 16.92 Å vacuum layer along the *c*-axis, and applying dipole correction. This structure fits the experimental results at room temperature (*13*). Similarly, U_{eff} of Cu was adjusted such that the qualitative orbital ordering of isolated CuPc is retained. The energy convergence criterion chosen for the self-consistency cycle was 1×10^{-7} eV. Ionic positions were optimized until the residual forces on each ion were less than 1×10^{-2} eV/Å^{-1}.

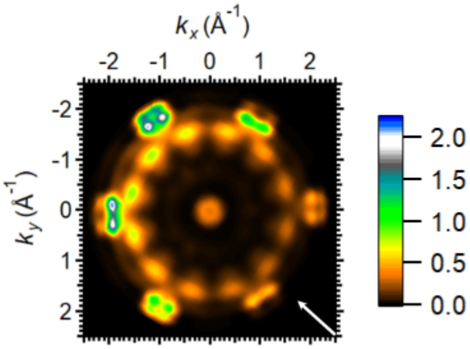

Supplementary Figure 11: Calculated momentum distribution at the CuPc HOMO energy for CuPc adsorbed in 3 rotational domains on top of a cluster of $TiSe_2$ in its normal phase. The white colored arrows indicate projected light incidence (315°).

We adopted a slab model with periodic boundary conditions, using a $TiSe_2$ supercell of 8x8x1 (a = b = 28.28 Å, c = 3.08 Å, $\alpha = \beta = 90°$ and $\gamma = 120°$), introducing a 16.92 Å vacuum layer along the c-axis, and applying dipole correction. The system comprised a single CuPc molecule adsorbed to this slab model. Since the CuPc molecules arrange in a point-on-line overstructure on top of $TiSe_2$, several adsorption sites and molecular in-plane rotations were

evaluated, all yielding similar results. During the geometry optimization, we allowed for relaxations of the atoms of the molecule as well as for the topmost layer of the $TiSe_2$ substrate. We also included dispersion corrections proposed by Grimme with PBE (PBE-D2) (*14*). From the structure optimization calculation, it was found that the adsorption distance between molecule and substrate is 3.16 Å.

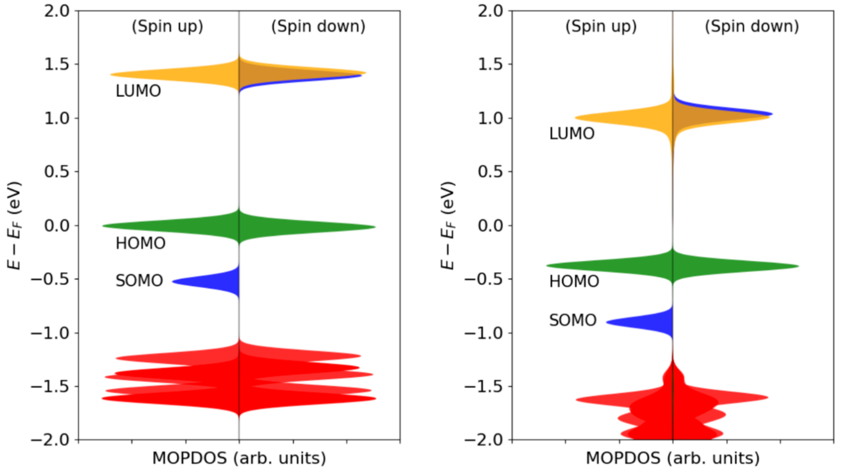

Supplementary Figure 12: MOPDOS of the isolated CuPc molecule (left) and of the CuPc molecule projected onto the band structure of an 8 x 8 x 8 Brillouin zones cluster of a $TiSe_2$ in its normal phase (right). The MOPDOS is individually plotted for the spin-up and spin-down components of an orbital. The LUMO (yellow), HOMO (green) and SOMO (blue) are colored individually and energetically deeper lying molecular orbitals are all colored in red.

The electronic structure of the adsorbed CuPc molecules was calculated using MOPDOS (*10*). In the left of Fig. 12 the energy levels for up-spin and down-spin orbitals are shown for an isolated molecule and on the right side the MOPDOS of the CuPc/$TiSe_2$ system is displayed, where the molecular orbitals are projected onto the DOS of the substrate. Both the orbital order and their relative energy differences as in the isolated molecule can be reproduced. Fig. 13 was unfolding E(K_i , E_m) picture into $E(k_i , E_m)$ picture for spin-up (left) and spin-down (middle) along the crystal directions marked in the primitive Brillouin zone of $TiSe_2$ (right). A wave vector "$\boldsymbol{K}$" of the supercell Brillouin zone is said to unfold into a wave vector "$\boldsymbol{k}$" in the primitive Brillouin zone. We used an unfolding technique that enabled the reconstruction of $E(\boldsymbol{k}, E)$ from a directly calculated E(K , E) (*15*). The horizontal axis is the $\boldsymbol{k}$ vector of the primitive Brillouin zone. The circle's radius in the figure indicates the magnitude of the spectral function $A(k_i, E_m)$ value for each k_i and E_m.

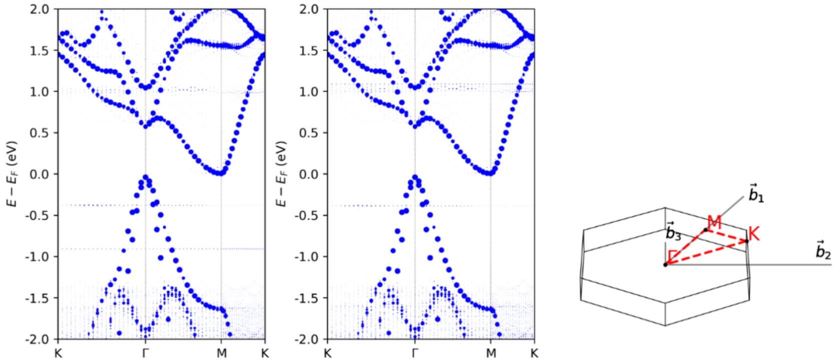

Supplementary Figure 13: The effective band structure for spin-up (left) and spin-down (middle) of CuPc/$TiSe_2$ is shown along the crystal directions as marked in the right. The respective unit cell vectors of the primitive Brillouin zone are labeled $\vec{\boldsymbol{b}}_1$, $\vec{\boldsymbol{b}}_2$ and $\vec{\boldsymbol{b}}_3$.

The Kohn-Sham orbitals of this calculation serve as the input for the subsequent simulation of the valence band momentum maps, which were calculated within the one-step model of photoemission (*16*) and for which we approximate the final state by a plane wave (*17*), (*10*). The sample geometry considers the experimental setup where *p*-polarized light is directed at the sample at an azimuthal incident angle of $\Phi = 315°$ (indicated by the white arrow) and at a polar incident angle of $\Theta = 68°$. The momentum map at the CuPc HOMO energy is calculated for a photoelectron kinetic energy of 29.4 eV.

The calculation of the C 1*s* core-level momentum distributions includes multiple scattering of the photoelectrons (*18*). This calculation method is based on Fermi's Golden rule and adopts the Lippmann Schwinger equation for the final state. The final state includes the Green's function and potential of the system, and it is possible to consider multiple scattering of photoelectrons in the system. In our calculation, both intramolecular scatterings of CuPc and scatterings at the substrate were considered. The atomic orbitals of C 1*s* orbitals calculated by Gaussian (*19*) represent the intial states. The same experimental geometry as in the valence band is applied for *p*-polarized XUV pulses with 86 eV. However, in the multiple scattering calculations, the substrate consists of a cluster of 58 atoms. We assumed 42 different geometrical models of CuPc and $CuPc^+$ atop $TiSe_2$ with varying molecular out-of-plane distortions, adsorption heights, adsorption sites and molecular in-plane rotations. For each model, we consider three molecular orientations along the substrate high symmetry directions according to the point-on-line growth and we add their intensities for the resulting XPD momentum map. The XPD momentum maps shown in the main manuscript sum over the intensities stemming from all carbon atoms that are

Orbital space	Ground state of CuPc	$CuPc^+$	Excited states of CuPc
Inactive	145	137	129
RAS1	0	8	16
Maximum number of holes	-	1	1
RAS2	4	4	4
RAS3	0	6	16
Maximum number of electrons	-	1	1

Supplementary Table 4: Numbers of orbitals in different groups in the RASSCF calculations of ground and excited states of neutral CuPc, and of $CuPc^+$. Inactive orbitals are the orbitals that are doubly occupied in all configurations. RAS1 orbitals are the orbitals that are doubly occupied except for a maximum number of holes allowed in this orbital subspace. RAS2 orbitals are the orbitals, for which all possible occupations are allowed. RAS3 orbitals are the orbitals that are unoccupied except for a maximum number of electrons allowed in this subspace.

located in the benzene rings. According to the experimental data treatment, the calculated data is three-fold symmetrized.

2.3 Calculations of the x-ray and XUV energy- and momentum-resolved signals from isolated CuPc and CuPc^+

2.3.1 Method

For the calculations of the excited and ground states of neutral and cationic CuPc, we use a multiconfigurational restricted active space self consistent field calculation (RASSCF) (*20*) implemented in the software program Molcas (*21*), with a truncated ANO-S basis set (*22*) for all atoms. Within the RASSCF method, molecular orbitals can be selected to be either always doubly occupied or can have various occupations in the configurations of the configuration-interaction (CI) expansion. We increase the number of the orbitals of the second type until the convergence is reached. The number of the corresponding orbitals of the second type are shown in Tab. 4. In Fig. 14, we show the RASSCF orbitals from the ground-state calculation of CuPc. They are similar to the orbitals computed with the DFT in the previous theoretical studies of CuPc (*23*–*25*).

We calculate the photoelectron probability depending on the photoelectron momentum $\mathbf{q}$

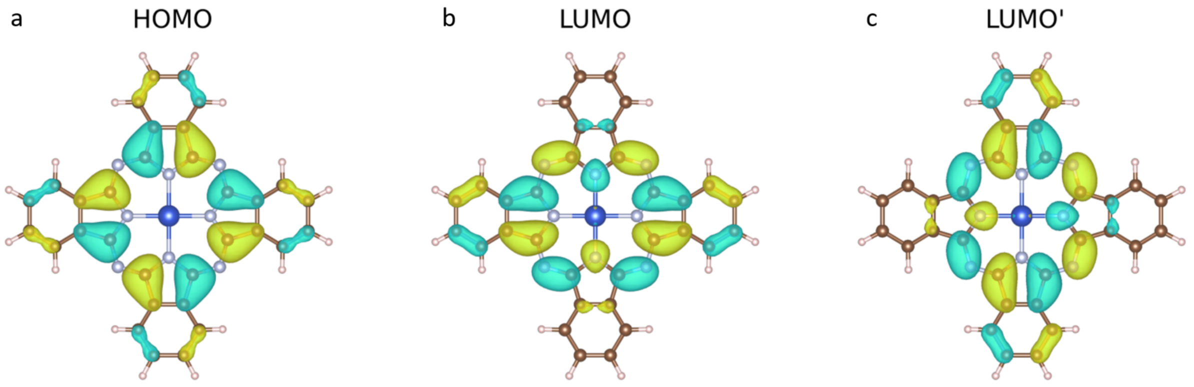

Supplementary Figure 14: RASSCF orbitals obtained in the ground-state calculation of neutral CuPc corresponding to (a) HOMO orbital, (b) LUMO orbital and (c) LUMO' orbital.

using the plane-wave approximation (PWA) to the photoelectron wave function as follows (*17, 26–28*)

$$P(\mathbf{q}) \propto |\boldsymbol{\epsilon}_{\text{in}} \cdot \mathbf{q}|^2 \sum_{F,\sigma} \delta_{\varepsilon_e, \omega_{\text{in}} - E_F^{N_{\text{el}}-1} + E_I^{N_{\text{el}}}} \left| \int d^3 r e^{-i\mathbf{q}\cdot\mathbf{r}} \phi_F^D(\mathbf{r})] \right|^2. \tag{2}$$

Here, $\phi_F^D(\mathbf{r})$ is a Dyson orbital, which is an overlap function between the N-electron wave function of an initial state Ψ_I^N, and $(N-1)$-electron wave function wave function, Ψ_F^{N-1}, produced by ionization

$$\phi_D(\mathbf{r}) = \sqrt{N} \int \Psi_I^N(\mathbf{r}_1, \cdots, \mathbf{r}_N) \Psi_F^{N-1}(\mathbf{r}_2, \cdots, \mathbf{r}_N) d\mathbf{r}_2 \cdots \mathbf{r}_N. \tag{3}$$

$E_I^{N_{\text{el}}}$ and $E_F^{N_{\text{el}}-1}$ are energies of states Ψ_I^N and Ψ_F^{N-1}, correspondingly. ω_{in} and ϵ_{in} are photon energy and the direction of the polarization vector of the photoionizing pulse, correspondingly. The summation in Eq. 2 runs over all possible final states F of the ionized system and indicates that one can detect a photoelectron with the energy ε_e, if the energy difference between N-electron and $N-1$ electron state match $\omega_{\text{in}} - \varepsilon_e$. We use atomic units for these and following expressions. In order to be able to compare our results with experimental data, we also three-fold symmetrize the calculated photoelectron momentum maps (see Section 1).

2.3.2 Molecular geometry

We take the ground-state geometry of neutral isolated CuPc from Refs. (*29, 30*). As described in the main manuscript, CuPc becomes positively charged right after time t_0. We assume that

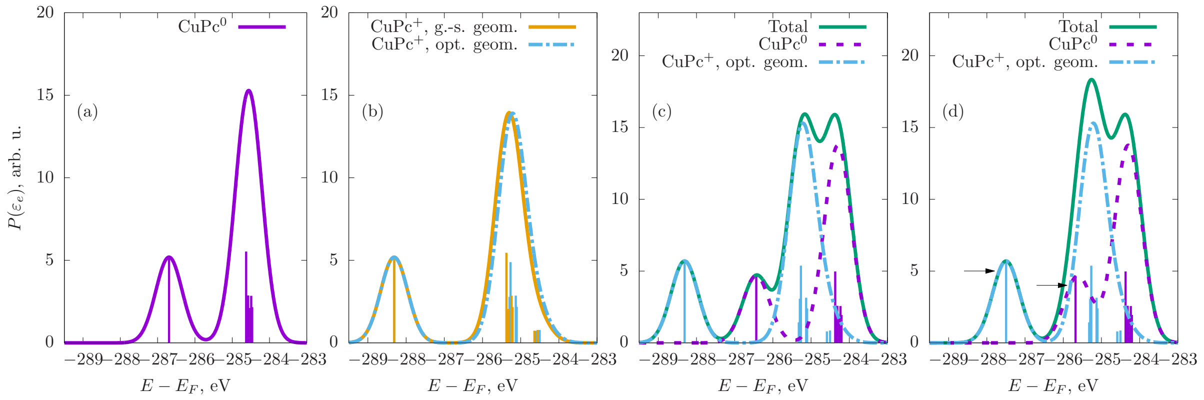

Supplementary Figure 15: (a) Simulated C 1s core level photoelectron spectra of neutral molecules, (b) ionized molecules in the ground-state geometry of the neutral state and the ground-state (”optimized”) geometry of the ionized state and (c) sum of the photoelectron spectra of neutral molecules and ionized molecules in the optimized geometry assuming their proportion of 45% to 55%, (d) the same as (c) except that the positions of the left peaks in the spectra of both neutral and ionized molecules are shifted by the same value of 820 meV. For the calculation of the sum, an additional shift of 260 meV was applied to the spectra from neutral molecules, which is due to the influence of the negatively-charged surface as determined in the experiment. Lines illustrate the intensities and positions of the individual peaks contributing to the spectra.

atoms of the molecule did not have time to move and the ionized molecule is in the ground-state geometry of the neutral state at the moment, when it gets positively charged. Since the ground-state geometry of the neutral state is not the ground-state geometry of the ionized state, atoms of a molecule would start moving towards the ground-state geometry of the cationic state. We suggest that this process happens until some time between t_1 and t_2. We find the ground-state geometry of an isolated cation $CuPc^+$ with molcas at the RASSCF level (*31*). The $CuPc^+$ should additionally move out of plane due to the interaction with a negatively-charged substrate. However, it is only computationally possible to find the optimized geometry of an isolated cation. Thus, we calculate signals from $CuPc^+$ in the ground-state geometry of the neutral state and in the optimized geometry of an isolated cation in order to compare with experimental data at different pump-probe delay times. We also calculate signals from excited and unexcited neutral CuPc in the ground-state geometry.

(a) (b) $\rho^{+}_{\text{g.-s. geom.}} - \rho^{0}_{\text{g.-s. geom.}}$ (c) $\rho^{+}_{\text{g.-s. geom.}} - \rho^{0}_{\text{g.-s. geom.}}$ (d) $\rho^{+}_{\text{opt. geom.}} - \rho^{+}_{\text{g.-s. geom.}}$

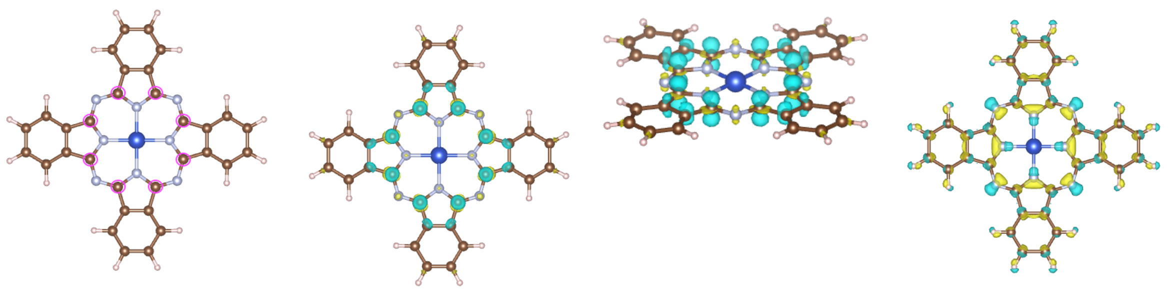

Supplementary Figure 16: (a) CuPc with outlined atoms, whose $1s$ core orbitals give rise to the left peak in the core spectra in Fig. 15. Density difference between (b), (c) ionized molecules and neutral molecules in the same geometry of the neutral ground state at different perspectives, and (d) between ionized molecules in the ground-state geometry of the neutral state and the ground-state ("optimized") geometry of the ionized state. Blue isosurface corresponds to the positive charge, yellow isosurface corresponds to the negative charge.

2.3.3 Core photoelectron spectra

Fig. 15 a shows the calculated C $1s$ core photoelectron spectrum of neutral CuPc. For the calculation of the C $1s$ core photoelectron spectra, we substituted the calculated molecular core orbitals for the Dyson orbitals $\phi^D_F(\mathbf{r})$ in Eq. 2 and averaged the probability $P(\mathbf{q})$ over the momentum angle at a fixed photoelectron energy $\varepsilon_e = |\mathbf{q}|^2/2$. In our calculation, the C $1s$ electrons belong to the inactive space and we obtain their orbital energies. They are approximately the binding energies with an addition of a correction due to electron correlations, which is approximately the same for the core orbitals in the calculation using the same RASSCF space (*32*). Since it is nontrivial to determine this correction, we adjust the position of the right intensive peak to agree with the position of the same peak in the experimental data in Fig. 2 c in the main text. For this and other photoelectron spectra in Fig. 15, we adjust the position of the right intensive peak to agree with the position of the same peak in the experimental data in Fig. 2 c. The resulting spectra are in a good agreement with the experimental data and are very similar to a previous theoretical calculations of core photoelectron spectra of neutral CuPc (*23*). In comparison with the experimental data, the position of the left small peak is shifted towards higher binding energies in the calculation and the ratio between the intensities of the two peaks is slightly different. The former is due to the contribution of the satellite peak of the right peak at the same position of the left peak, which makes it more intense in the experiment (*23*). Since we do not model the satellite peaks in our calculations, we cannot reproduce this intensity in-

crease. We find that the left peak is due to Carbon atoms in the main ring around Copper (see Fig. 16 a), and the right peak is due to Carbon atoms in the benzene rings.

The disagreement of the position of the left peak is due to the approximation of binding energies as the molecular orbital energies. Ideally, we should have compared the energy difference between CuPc with a hole in a $1s$ shell of a carbon atom and neutral CuPc. The hole in a C $1s$ shell would lead to changes of the energies in the valence state of CuPc that would depend on the exact position of the Carbon atom with a hole. These site-specific changes would lead to the adjustments of the relative positions of the peaks. Unfortunately, a calculation of CuPc with a core hole at a single site is almost impossible, because we would not be able to make use of the symmetry of the molecule in this case and the already challenging computational requirements would increase by several orders.

With our approach, we are able to calculate the core photoelectron spectra for $CuPc^+$ with the positive charge in the valence states. The spectra before and after the geometry optimization of $CuPc^+$ are shown in Fig. 15 b. We adjust the position of the right peak of the spectra from $CuPc^+$ in the optimized geometry to the experimental position. We apply the same shift to the spectra from molecules in both geometries and observe that the right peak has slightly shifted towards lower binding energies after the geometry optimization.

Let us compare the spectra of CuPc and $CuPc^+$ in Figs. 15 a and b. The spectra of $CuPc^+$ have shifted towards higher binding energies after ionization, which is due to reduced Coulomb screening after removing one electron from CuPc. Thereby, the right peak has shifted by 0.6 eV and the left peak has shifted by 1.6 eV. The separation between the left and the right peak increased by 1 eV. Although, the binding energy of the left peak is overestimated in our calculation, we reproduce the increase of the separation of the right and left peaks by 1 eV after creation of a positive charge in the experimental data in Fig. 2 c in the main text. We also find that the right peak gets broader by 4% and, consequently, its intensity decreases.

In order to understand the changes in the spectra, we plot the difference between the electron density of $CuPc^0$, $\rho^0_{\text{g.-s. geom.}}$, and the electron density of $CuPc^+$, $\rho^+_{\text{g.-s. geom.}}$, in the same geometry in Fig. 16 b and c. It follows from this plot that the positive charge (blue isosurface) is mainly localized around carbon atoms in the central ring and they experience much larger local decrease of Coulomb screening than other carbon atoms, which leads to a larger shift of the left peak in the core photoelectron spectra. Carbon atoms in the benzene ring that are nearest neighbours to carbon atoms in the main ring also experience a larger decrease of Coulomb screening than other carbon atoms in the benzene ring. Therefore, the shift of their binding energies is larger

than of the other benzene carbon atoms, which explains the broadening of the right peak.

Fig. 16 d shows the difference between the electron densities of $CuPc^+$ before and after the optimization of their geometry, $\rho^+_{\text{g.-s. geom.}}$ and $\rho^+_{\text{opt. geom.}}$, respectively. The difference is mainly determined by the change of atomic positions. When an atom has moved in a certain direction, the density difference shows the excess of the negative charge (yellow isosurface) at the new position and deficiency of the negative charge (blue isosurface) at the former position. Since this effect is dominant for the density difference, it is hard to capture charge rearrangements relative to atomic positions from this plot. This density difference is, thus, very illustrative to follow the change of atomic positions. We observe that nitrogen atoms have the largest position changes among other atoms. Carbon atoms in the main ring move towards copper, and the benzene rings get more separated from the main ring.

In Fig. 15 c, we add the core photoelectron spectra in a and b assuming the proportion of 45% to 55% of neutral to positively-charged molecules. For the sake of comparison with the experiment, we also apply an additional shift of 260 meV to the spectra of neutral CuPc, which results due to the influence of the surface potential as described in the main text. We obtain a quite similar structure of the spectrum as in the experiment in Fig. 2 c in the main text. The right peak is split into the two peaks due to different contributions of $CuPc^0$ and $CuPc^+$. The proportion of the intensities of the two peaks deviates from the experiment, because we do not reproduce the position of the left peak exactly. The left peak of $CuPc^0$ overlaps with the right peak of $CuPc^+$ in the experimental data and contributes to the intensity of the corresponding sub-peak of the right peak.

In order to be able to compare the spectra with both contributions of $CuPc^0$ and $CuPc^+$ with the experimental ones, and knowing that the position of the left peak is shifted by $\sim$ 800 meV compared to the experiment, we shift the positions of the left peaks in the spectra of both $CuPc^0$ and $CuPc^+$ by the same value of $\sim$ 800 meV. The resulting spectra in Fig. 15 d are in a perfect agreement with the experiment.

We had to adjust the positions of the peaks in the core photoelectron spectra in order to get a perfect agreement with the experimental spectra. Still, several important results concerning the change of the spectra due to contribution of $CuPc^+$ were independent of this fitting and agree with the experiment. We reproduce the increase of the splitting between the right and the left peak in the spectra of $CuPc^+$ by about 1 eV, the broadening of the right peak and its intensity decrease. We find that the changes of the core photoelectron spectra are mainly explained by the appearance of the positive charge in the valence states of $CuPc^+$ and are not notably influenced

by structural rearrangements. Thus, our study strengthens the conclusion that the ratio between CuPc and $CuPc^+$ can be determined from the core photoelectron spectra.

2.3.4 Occupation of LUMO and LUMO' orbitals

We perform the calculation of the excited states of neutral CuPc and find that the first two excited states are nearly degenerate. Depending on the symmetry restriction in the simulation, the two states are split in energy by either $0.07\ \mathrm{meV}$ ($\mathrm{D_{2h}}$-symmetry) or $13\ \mathrm{meV}$ ($\mathrm{C_{2h}}$-symmetry). The dominant contribution to the first excited state is given by a configuration with a singly occupied HOMO and a singly occupied LUMO, and the dominant contribution of the second excited state is given by a configuration with a singly occupied HOMO and a singly occupied LUMO' orbitals. We find that there is only one possible final ionized state Ψ_F^{N-1} that can be reached by the ionization of CuPc being in the first excited and being in the second excited state [see Eq. (2)]. This state has a dominant contribution from a configuration with a singly occupied HOMO and unoccupied LUMO and LUMO'. Thus, the Dyson orbital for the case of any of the two excited states is simply given by a corresponding LUMO or LUMO' orbital.

We first perform a calculation of PMMs corresponding to the excited state assuming the mechanism of intramolecular photoexitation. The assumption of intramolecular photoexcitation implies that LUMO and LUMO' orbitals of CuPc adsorbed on $TiSe_2$ are also degenerate. We find that resulting PMM has three-fold rotational symmetry, whereas the experimental PMM has six-fold rotational symmetry (see Fig. 1 c first row of the main manuscript and Fig. 18 c below). The mismatch between the calculation and the experimental data excludes the mechanism of the intramolecular photoexcitation.

Due to the interaction with a substrate, the characteristics of the excited states of a molecule can change, and the degeneracy between LUMO and LUMO' orbitals can be lifted. The interaction can also lead to the emergence of new molecular orbitals that are combinations of LUMO and LUMO', because a certain symmetry of the molecular excited states can become more favorable due to the symmetry of the substrate.

We calculate the PMM under the assumption that one molecular orbital

$$\phi_{L_1}(\mathbf{r}) = \sin\alpha\,\phi_{L_x}(\mathbf{r}) + \cos\alpha\,\phi_{L_y}(\mathbf{r}), \tag{4}$$

got occupied after photoexcitation. Here, $\phi_{L_x}(\mathbf{r})$ and $\phi_{L_y}(\mathbf{r})$ are LUMO and LUMO' orbitals. α is a parameter that determines the coefficients of the linear combination. We calculate PMMs for different values of the parameter α shown in Fig. 17. We obtain that all PMMs have six-

fold symmetry in agreement with the experiment. The PMM in Fig. 17 d that assumes $\alpha = \pi/6$ is in an excellent agreement with the experiment. Fig. 18 a shows the orbital $\phi_{L_1}(\mathbf{r}) = (1/2)\phi_{L_x}(\mathbf{r}) + (\sqrt{3}/2)\phi_{L_y}(\mathbf{r})$ obtained for $\alpha = \pi/6$ in real space. Fig. 18 b shows the PMM for $\alpha = \pi/6$ in front of the PMM obtained from the measured data interpolated by a Gaussian filter in Fig. 18 c in order to highlight the excellent agreement. This confirms that the PMM right after photoexcitation corresponds to occupation of LUMO and LUMO' orbitals.

2.3.5 Photoelectron momentum maps due to the HOMO orbital

Within the RASSCF method, the Dyson orbital is as a linear combination of molecular orbitals that are occupied in configurations entering Ψ_I^N and are unoccupied in configurations entering Ψ_F^{N-1} in Eq. (2) (*28*). We calculate and analyze possible final states after ionization of neutral CuPc by a probe pulse, and obtain that the Dyson orbital corresponding to the PMM from neutral CuPc in Fig. 1 c, third row in the main text is given by

$$\phi_F^D(\mathbf{r}) \propto -0.890\phi_H(\mathbf{r}) - 0.198\phi_{H-2}(\mathbf{r}), \tag{5}$$

where $\phi_H(\mathbf{r})$ and ϕ_{H-2} are the HOMO and HOMO-2 orbitals correspondingly. We calculate the PMM substituting the above expression for the Dyson orbital and compare it to the PMM under the assumption that the Dyson orbital is simply the HOMO orbital. We find that the PMMs do not considerably differ.

We now consider PMM from $CuPc^+$. In our calculations, we apply an orbital optimization procedure, and the shapes of molecular orbitals of $CuPc^+$slightly differ from the shapes of molecular orbitals of neutral CuPc. We obtain that the configuration with a singly occupied HOMO orbital is dominating for the ground state of $CuPc^+$, which is the state Ψ_I^{N-1} in Eq. (2). In order to obtain Ψ_F^{N-1} in Eq. (2), we need to calculate the doubly ionized state of CuPc. Unfortunately, this is computationally too demanding and we have to approximate the Dyson orbital by the HOMO orbital for the calculation of the PMM.

In order to compare the PMM from CuPc and $CuPc^+$, we have to assume that the Dyson orbital is the HOMO orbital in both the cases. To compute results we use the structural parameters for the neutral CuPc and the $CuPc^+$ extracted from the experiment. The structural parameters for the $CuPc^+$ take into account the out-of plane atomic rearrangements which occur due to the interactions between the positively charged CuPc and the negatively charged substrate. These PMM results are shown in Figs. 19 a for CuPc and Figs. 19 b for $CuPc^+$. These simulated results match closely with the experimental PMMs given in Fig. 1 c, third row in the main text. We find

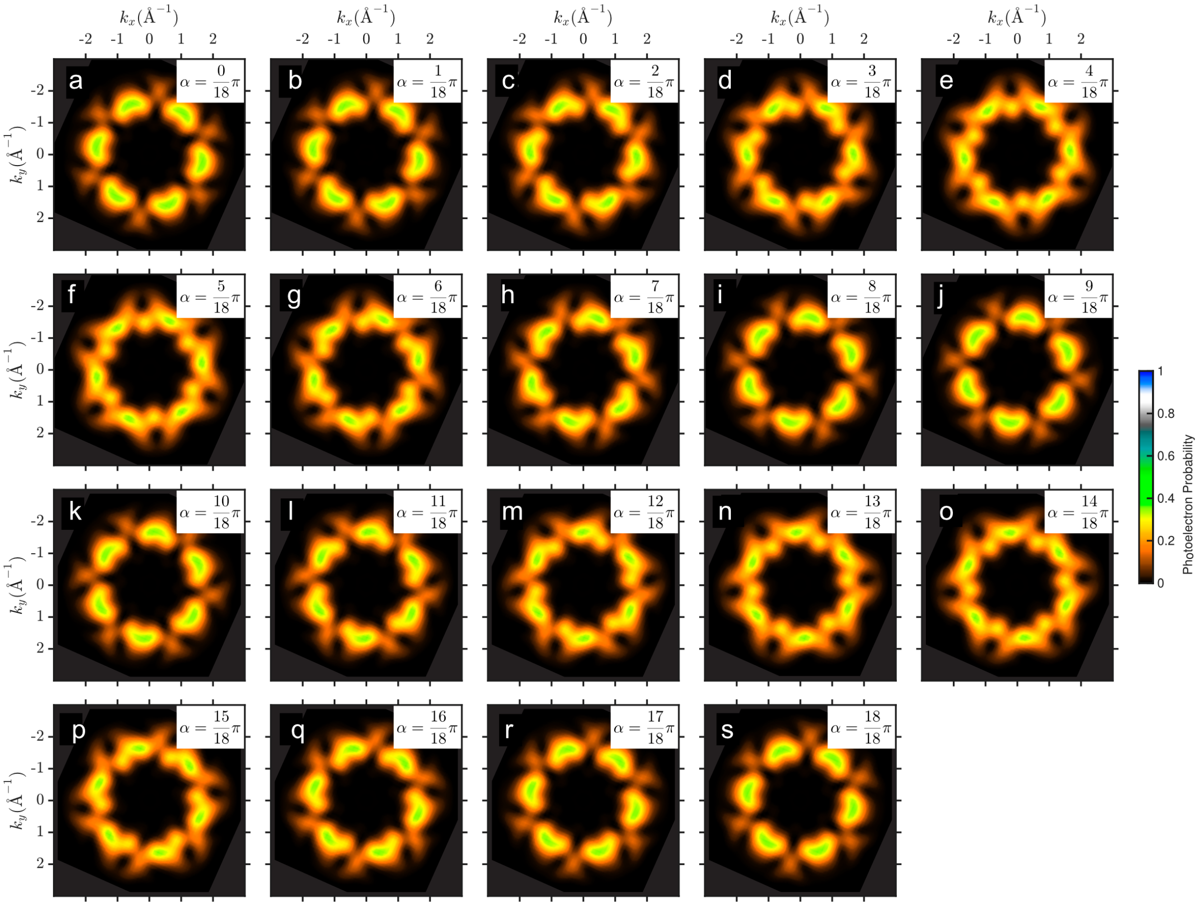

Supplementary Figure 17: PMMs under the assumption that the orbital $\sin\alpha\,\phi_{L_x}(\mathbf{r}) + \cos\alpha\,\phi_{L_y}(\mathbf{r})$ gets occupied. The values of the parameter α are (a) $\alpha = 0$, (b) $\alpha = \frac{1}{18}\pi$, (c) $\alpha = \frac{2}{18}\pi$, (d) $\alpha = \frac{3}{18}\pi$, (e) $\alpha = \frac{4}{18}\pi$, (f) $\alpha = \frac{5}{18}\pi$, (g) $\alpha = \frac{6}{18}\pi$, (h) $\alpha = \frac{7}{18}\pi$, (i) $\alpha = \frac{8}{18}\pi$, (j) $\alpha = \frac{9}{18}\pi$, (k) $\alpha = \frac{10}{18}\pi$, (l) $\alpha = \frac{11}{18}\pi$, (m) $\alpha = \frac{12}{18}\pi$, (n) $\alpha = \frac{13}{18}\pi$, (o) $\alpha = \frac{14}{18}\pi$, (p) $\alpha = \frac{15}{18}\pi$, (q) $\alpha = \frac{16}{18}\pi$, (r) $\alpha = \frac{17}{18}\pi$, (s) $\alpha = \frac{18}{18}\pi$. All PMMs have been three-fold symmetrized.

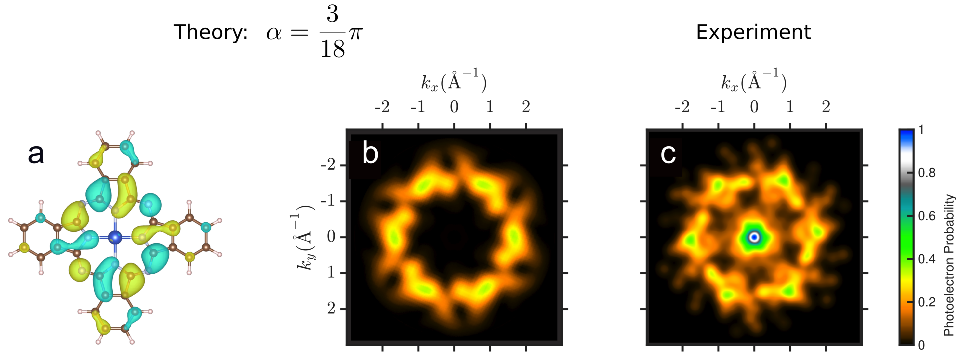

Supplementary Figure 18: (a) The orbital $\phi_{L_1}(\mathbf{r}) = (1/2)\phi_{L_x}(\mathbf{r}) + (\sqrt{3}/2)\phi_{L_y}(\mathbf{r})$ in real space and (b) PMM under the assumption of the occupation of this orbital. (c) Experimental PMM obtained from the measured data interpolated by a Gaussian filter. The PMMs have been threefold symmetrized.

that the ionization of CuPc leads to an instantaneous significant change in the PMMs. We obtain that the PMM ring resulting from the neutral CuPc in Figs. 19 a has a radius of $k_{max} = 1.484$ Å^{-1}. While the PMM ring resulting from CuPc^+ after atomic rearrangements in Figs. 19 b has a radius $k_{max} = 1.479$ Å^{-1}, which is 0.4% smaller than in Figs. 19 a. The width of the PMM ring shows an 1% increase from 0.363 Å^{-1} for CuPc neutral to 0.367 Å^{-1} for CuPc^+. Here we obtain a similar trend as in the experiment where the ring radius decreases and the ring width increases (see Fig. 3 e). The quantitative difference between the theoretical and the experimental results could be because we calculate the PMM for isolated CuPc molecule and the interactions between the CuPc molecule and the substrate are not accounted for in the electronic structure and the PMM calculations.

2.4 Pair-potential calculation

From the analysis of the x-ray photoemission spectra, we obtained that nearly half of CuPc molecules become positively ionized in 375 fs after the excitation. We investigate the influence of ionization of the adsorbed molecules on their orientation by means of a pair-potential model as described in the work by Kröger et al. (*33*). This pair-potential model has been successfully applied for the description of the geometry of several organic thin films on a substrate (*33*–*37*). Consider two isolated molecules A and B, the pair-potential between molecule A with atoms i

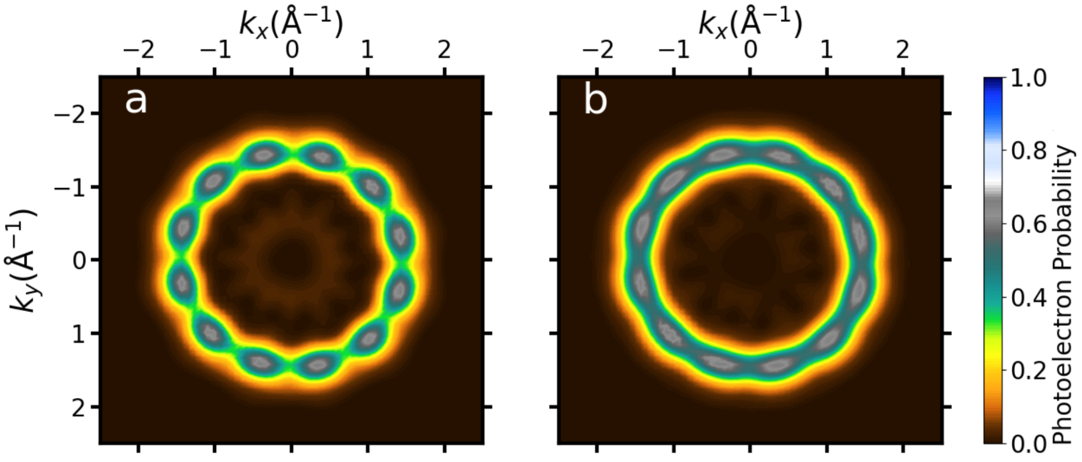

Supplementary Figure 19: PMMs from (a) neutral CuPc, and (b) $CuPc^+$. The PMMs have been three-fold symmetrized.

and the molecule B with atoms j is

$$\phi_{AB} = \sum_{i,j} \left(\phi_{ij}^{vdW}(r_{ij}) + \phi_{ij}^{El}(r_{ij}) \right), \tag{6}$$

where $\phi_{ij}^{vdW}(r_{ij})$ is the van der Waals and $\phi_{ij}^{El}(r_{ij})$ is the electrostatic potential, which both depend on the distance r_{ij} between atoms i and j. The electrostatic potential depends on the partial charges Z_i and Z_j of the atoms i and j

$$\phi_{ij}^{El}(r_{ij}) = \frac{Z_i Z_j}{4\pi\epsilon_0 r_{ij}}. \tag{7}$$

In the RASSCF calculations in Sec. 2.3, the computed molecular states and the molecular orbitals are constructed from a basis of atomic orbitals. Therefore, we obtain the partial charges at each atom from the calculated molecular states. The partial charges for isolated neutral and ionized CuPc are shown in Tab. 5, with the corresponding atomic labels in Fig. 20.

The van der Waals potential includes an exponential Pauli repulsion and an attractive London force as

$$\phi_{ij}^{vdW}(r_{ij}) = a_{ij} \exp(-b_{ij} r_{ij}) - c_{ij} r_{ij}^{-6}, \tag{8}$$

where the coefficients a_{ij}, b_{ij} and c_{ij} depend on the specific element of the atoms i and j. We take these parameters from Ref. (*21*).

We determine the orientation of the molecules before and after excitation by finding molecular orientations that lead to the minimum of the pair-potential. Before the sample is excited,

atom	$CuPc^0$	$CuPc^+$
C1	$+0.2293$	$+0.2730$
C2	$+0.1246$	$+0.1310$
C3	-0.1906	-0.1838
C4	-0.1471	-0.1297
H1	$+0.1611$	$+0.1775$
H2	$+0.1437$	$+0.1664$
N1	-0.4289	-0.4119
N2	-0.4350	-0.4290
Cu	$+0.8873$	$+0.8887$

Supplementary Table 5: Partial charges at each atom for different states of CuPc. The atom label in the molecule is shown in Fig. 20.

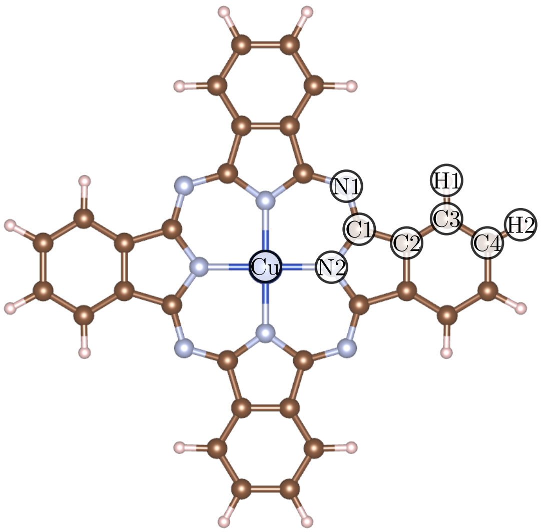

Supplementary Figure 20: Atomic structure of the CuPc molecule with the atomic labels used for the description of the partial charges in Tab. 5

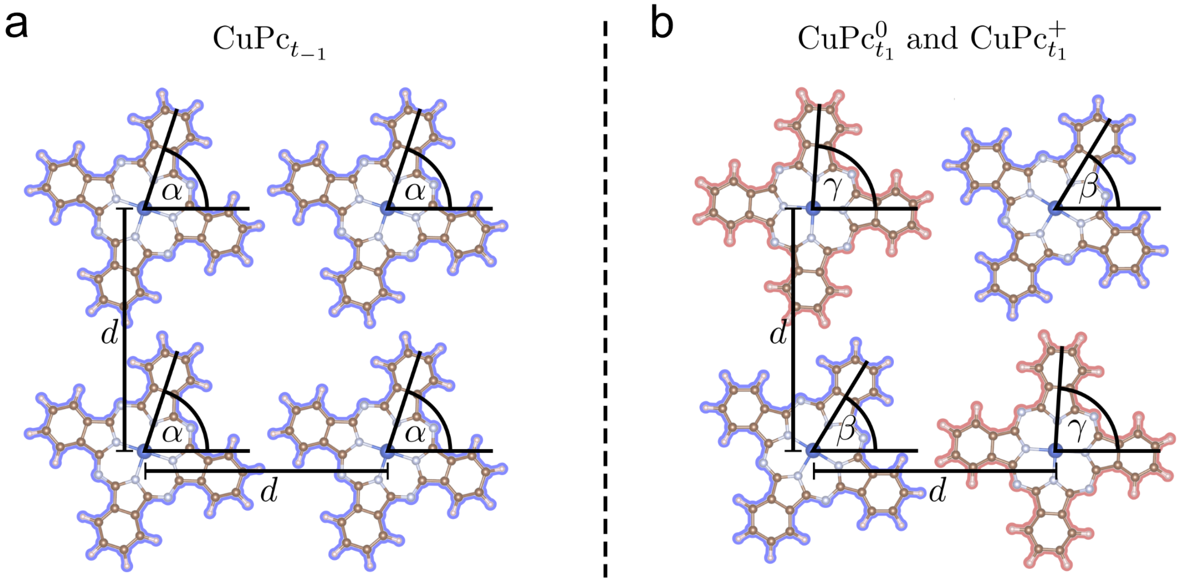

Supplementary Figure 21: Visualization of the two-dimensional grid of the molecules used in the pair-potential calculation. (a) shows the model of the nonexcited molecular layer containing neutral molecules (blue). (b) shows the model of the molecular layer after excitation with neutral molecules (blue) and singly ionized ones (red) ordered in a checkerboard pattern.

the molecules on the substrate are neutral. This corresponds to the molecular layer at the timep t_{-1}, which we refer to as the nonexcited molecular layer. We model it with a two-dimensional layer of $\mathrm{CuPc}_{t_{-1}}$ molecules. The LEED measurement of the molecular film before the excitation shows a highly ordered film, meaning that all molecules have the same alignment relative to each other. The experimental photoelectron momentum map corresponding to the HOMO orbital of $\mathrm{CuPc}_{t_{-1}}$ shown in the main manuscript also indicates that all molecules have the same orientation. Therefore, we assume that the angle α between the molecular axis and the vector connecting the centers of the nearest-neighbor molecules along the horizontal direction is the same for all molecules. The structure simulating the nonexcited molecular layer is shown in Fig. 21 a.

The molecular layer after the excitation at the time t_1, when about half of the molecules are ionized, is simulated by the structure, in which every neutral CuPc molecule has a positively charged neighbor and vice versa as shown in Fig. 21 b. This results in a layer of molecules that is ordered like a checkerboard pattern of ionized and neutral molecules. We assume that the angle β between the molecular axis of $\mathrm{CuPc}^0_{t_1}$ and the vector connecting the centers of the nearest-neighbor molecules in the horizontal direction is the same for all neutral molecules

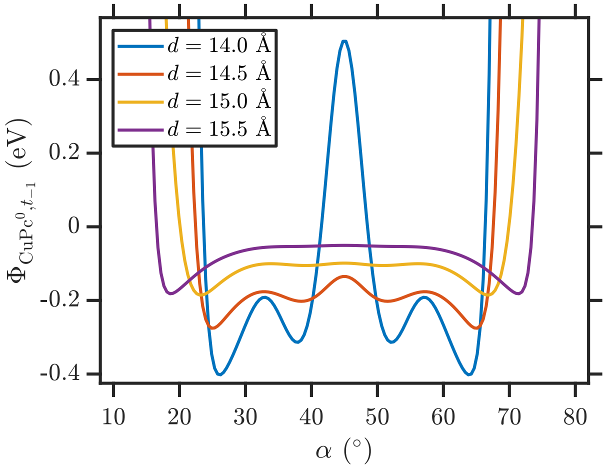

Supplementary Figure 22: Pair-potential $\Phi_{\mathrm{CuPc}^0,t_{-1}}$ as the function of the orientation angle α calculated for the nonexcited molecular layer at different distances d between the molecules.

$\mathrm{CuPc}^0_{t_1}$. Similarly, we assume that the angle γ, which is the angle between the molecular axis of $\mathrm{CuPc}^+_{t_1}$ and the vector connecting the centers of the nearest-neighbor molecules in the horizontal direction, is the same for all $\mathrm{CuPc}^+_{t_1}$.

In both model structures, we neglect any shifting or bending of the molecular structure to the surface and place all molecules in one layer. This is justified by the results of the DFT calculations showing that the atomic displacement perpendicular to the surface for the closest hydrogen atoms is less than 0.2 Å. This means that a maximal change in the distance between the atoms r_{ij} is 2% for the closest hydrogen atoms. For the closest carbon atom, it is below 1% and for all the other atoms it is less.

We consider two sizes of model structures. In one case, the model structure consists of five molecules: a molecule in the center and its four nearest-neighbor molecules. In the other case, the model consists of 13 molecules: a molecule in the center, its nearest, next-nearest, and second next-nearest neighbors. As we will show below, our conclusions do not depend on the size of the structures. If not stated otherwise, the results are shown for the smaller structure.

We first consider the case of the nonexcited molecular layer. We show the pair-potential as a function of the angle α at different distances d between the nearest neighbor molecules in Fig. 22. The angle that minimizes the pair-potential, which we refer to as ”the optimal angle”, should provide the initial orientation of the molecules before the photoexcitation. We find that this angle depends on the distance between the molecules. This means that the orientation of molecules in the molecular layer depends on the distance between the molecules. We then

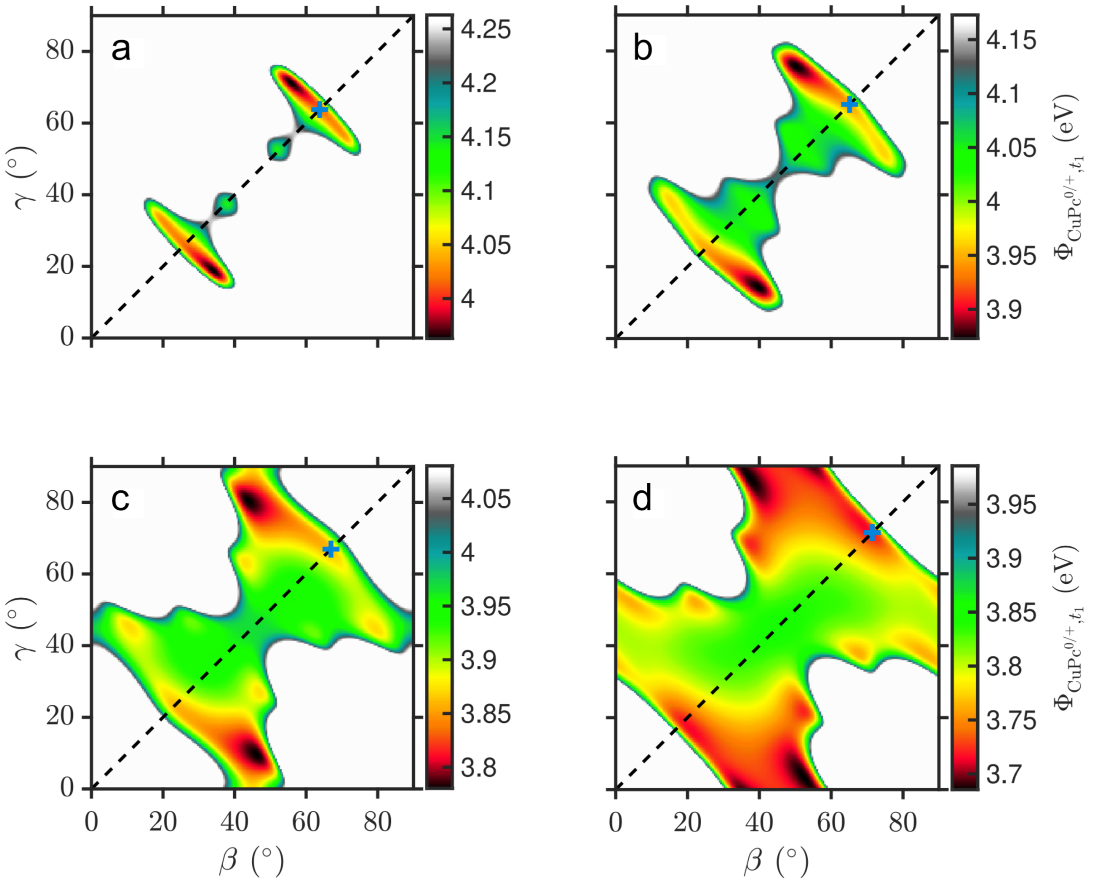

Supplementary Figure 23: Pair-potential $\Phi_{\text{CuPc}^{0/+},t_1}$ as a function of the orientation angles β and γ calculated for the excited molecular layer. The pair-potential is shown for (a) $d = 14.0$ Å, (b) $d = 14.5$ Å, (c) $d = 15.0$ Å and (d) $d = 15.5$ Å, where d is the distance between next-neighbor molecules. The black dotted line marks the values where both angles are equal $\beta = \gamma$. The blue plus in the plots marks the angle α which minimizes the pair-potential for the nonexcited molecular layer.

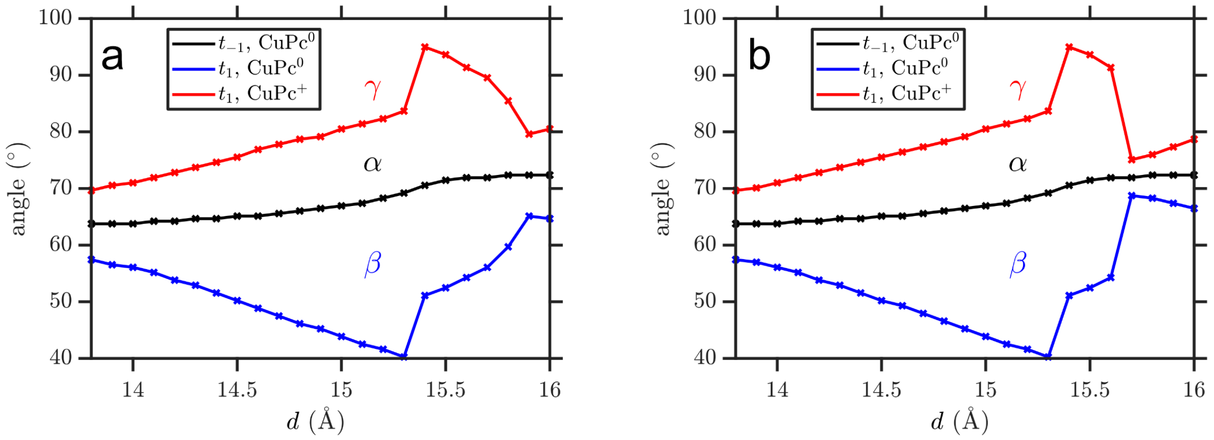

Supplementary Figure 24: Angles α, β, γ which minimizes the pair-potential depending on the distance d between the molecules for different structures. The black line corresponds to the optimal angle α in the model of the nonexcited molecular layer shown in Fig. 21 a. The blue and red lines show the optimal angles β and γ in the model of the molecular layer after excitation shown in Fig. 21 b. In (a) the calculation is obtained from a molecular layer that includes a total of 5 molecules, where one neutral-charged molecule is in the middle and the pair-potential is calculated by considering only nearest neighbor molecules. The result in (b) is obtained from a molecular layer that takes the nearest, next-nearest, and second-next-nearest neighboring molecules into account leading to the total of 13 molecules.

consider the molecular layer after excitation and calculate the pair-potential as a 2D function of the angles β and γ at different distances, which is shown in Fig. 23 at selected distances. The pair of angles (β, γ) that minimizes the potential describes the rotation of neutral and positively charged molecules after the excitation.

We find that the optimal angles in the case of the layer after excitation are different from the optimal angle in the case of the nonexcited layer. This means that both $\mathrm{CuPc}^0_{t_1}$ and $\mathrm{CuPc}^+_{t_1}$ rotate relative to their initial positions after the photoexcitation as a result of the emergence of the positive charge on half of the molecules.

The optimal angles α for the case of the nonexcited structure, are shown in the black line in Fig. 24 a as a function of the distance between the nearest neighbor molecules. The optimal angles β and γ for the case of the structure after excitation as a function of the distance between the molecules are shown in blue and red lines in Fig. 24 a, correspondingly. Up to the intermolecular distance of about 15.3 Å, these dependencies have the same trend. The neutral molecules rotate clockwise and the ionized molecules rotate anti-clockwise relative to the position of CuPc molecules in the nonexcited layer. The angles of rotation of neutral and ionized

CuPc relative to their initial positions are similar at smaller distances and are $(\beta - \alpha) = -7.7°$ and $(\gamma - \alpha) = 7.2°$ at $d = 14.0$ Å. With increasing distance, the angles of rotation increase and this increase is faster for neutral molecules. At $d = 15.0$ Å, the angles of rotation are $\beta - \alpha = -23.1°$ and $\gamma - \alpha = 13.6°$. At distances larger than 15.3 Å, the pair-potential as a function of the orientation angles starts to become flat and several local minima appear. Which local minimum becomes a global minimum starts to be quite sensitive to the geometry of the layer. For this reason, the optimal angles as the function of the distance are not a continuous function anymore. The pair-potential becoming flat at these distances and the appearance of several local minima are the indications that the interaction between the molecules becomes negligible. Thus, the charging of the molecules would not cause the unified rotation of the molecules as observed in the experiment, if the intermolecular distance were larger than 15.3 Å.

Due to the symmetry of the molecules, the potential has two optimal angles α and $90° - \alpha$ in the range $\alpha \in [0°, 90°]$ for the case of the nonexcited layer. Similarly, the potential has two pairs of optimal angles (β, γ) and $(90° - \beta, 90° - \gamma)$ in the range $\beta, \gamma \in [0°, 90°]$ for the case of the layer after excitation. Since it would not be realistic that the molecules rotate by more than 45°, we compared the optimal angle before the excitation with the pair of optimal angles (β, γ) after excitation that are closest to it. However, if we assume the second optimal angle for the nonexcited molecular layer $90° - \alpha$ as the initial angle of alignment and compare it to the closest pair $(90° - \beta, 90° - \gamma)$ for the layer after excitation, we find that neutral molecules would rotate anti-clockwise and ionized molecules would rotate clockwise relative to the initial alignment. These rotation directions are opposite to those obtained experimentally, so for the analysis we focus on the optimal angle α for the molecules in the nonexcited layer.

In Fig. 24 b we show the optimal angles as a function of distance for the structure of 13 molecules in order to compare it with the results for the structure consisting of five molecules. We find that results do not depend on the size of the structure up to the distance of 15.6 Å. The possible explanation for the difference at larger distances than 15.6 Å is that the interaction between the molecules becomes smaller and the pair-potential as a function of angles is approximately flat around the optimal angle. We can also see that the potential becomes flatter for higher distances by comparing the potentials for different distances in Fig. 23. Therefore a slight change in the potential due to interaction of molecules at distances beyond next neighbors leads to a change in the optimal angle.

Finally, we address the influence of the substrate on the presented results. In the case of the nonexcited structure, the interaction between molecules and substrate would lead to an

additional term in the pair-potential

$$\Phi_{total,t_{-1}} = \Phi_{M\leftrightarrow M,t_{-1}} + \Phi_{M\leftrightarrow S,t_{-1}}, \tag{9}$$

where $\Phi_{M\leftrightarrow M,t_{-1}}$ is the pair-potential between all CuPc molecules and $\Phi_{M\leftrightarrow S,t_{-1}}$ is the pair-potential between each CuPc molecule and the substrate. $\Phi_{M\leftrightarrow S,t_{-1}}$ depends on the distance between molecules and the substrate and it also depends on the relative position of the molecules to the substrate, which results in the domain structures. However, the dependence of the potential $\Phi_{M\leftrightarrow S,t_{-1}}$ on the angle α should not be pronounced, since the LEED data shows that molecules are oriented equally relative to each other. Thus, we assume that $\Phi_{M\leftrightarrow S,t_{-1}}$ as a function of α is constant.

In the case of the structure after excitation, about half of the molecules become positively charged and the substrate becomes negatively charged, which increases their interaction and may have an influence on angles β and γ. The pair-potential for the molecular layer and substrate after excitation is

$$\Phi_{total,t_1} = \Phi_{M\leftrightarrow M,t_1} + \Phi_{M\leftrightarrow S,t_1}, \tag{10}$$

where $\Phi_{M\leftrightarrow M,t_1}$ is the pair-potential between all the CuPc molecules and $\Phi_{M\leftrightarrow S,t_1}$ between the CuPc molecules and the substrate. We can rewrite it as

$$\Phi_{total,t_1} = \Phi_{M\leftrightarrow M,t_1} + \Phi_{M\leftrightarrow S,t_1} - \Phi_{M\leftrightarrow S,t_{-1}} + \Phi_{M\leftrightarrow S,t_{-1}} \tag{11}$$

Since we assumed that $\Phi_{M\leftrightarrow M,t_{-1}}$ is constant as a function of α, we consider the consequence of

$$\Phi_{M\leftrightarrow S,t_1} - \Phi_{M\leftrightarrow S,t_{-1}} = \underbrace{\Phi^{vdW}_{M\leftrightarrow S,t_1} - \Phi^{vdW}_{M\leftrightarrow S,t_{-1}}}_{\Delta\Phi^{vdW}_{M\leftrightarrow S,t_1,t_{-1}}} + \underbrace{\Phi^{El}_{M\leftrightarrow S,t_1} - \Phi^{El}_{M\leftrightarrow S,t_{-1}}}_{\Delta\Phi^{El}_{M\leftrightarrow S,t_1,t_{-1}}} \tag{12}$$

for the minimum of the pair-potential as a function of β and γ, where we inserted the corresponding van der Waals and electrostatic potential explicitly.

From the experimental results showed in the main manuscript, we obtain that the negative charge in the substrate is distributed in the 3d band of the Titanium atoms. Thus, we approximate $\Delta\Phi^{El}_{M\leftrightarrow S,t_1,t_{-1}}$ by the electrostatic potential describing the interaction between molecules and negatively charged Titanium atoms. The bending of the molecules from the plane after photoexcitation leads to the change of the distance between molecular and substrate atoms and also influences $\Delta\Phi^{El}_{M\leftrightarrow S,t_1,t_{-1}}$, which we assume to be negligible as a function of the orientation angles. We also assume that the bending of the molecules from the plane does not affect the

van der Waals potential, therefore $\Delta\Phi^{vdW}_{M\leftrightarrow S,t_1,t_{-1}}$ is also assumed to be negligible as a function of the orientation angles. Thus, we determine the optimal angles for the excited structure as the minimum of the pair-potential

$$\Phi_{total,t_1}(\beta,\gamma) \approx \Phi_{M\leftrightarrow M,t_1} + \Delta\Phi^{El}_{M\leftrightarrow S,t_1,t_{-1}} + \text{Const.} \tag{13}$$

The charge in all Titanium atoms in the upper layer of the substrate is chosen such that it compensates for the positive charge of the molecules. We compare the results at four specific distances 14.0 Å, 14.5 Å, 15.0 Å, 15.5 Å. We plot the pair-potential of the structure after excitation taking the negatively charged Ti atoms into account as a function of angles β and γ in Fig. 25. Let us compare it with the potential in Fig. 22, which includes only the interaction between molecules. The term $\Delta\Phi^{El}_{M\leftrightarrow S,t_1,t_{-1}}$ contributes to a constant shift of the potential. However, we find that it does not result in the change of the overall structure of the potential and the angles β and γ, when the potential has a minimum, are the same for all shown distances. Thus, we obtain that the negative charge in the substrate does not have an effect on the alignment of the molecules relative to each other.

With the pair-potential calculation, we identified that the rotation of the molecules after the excitation is caused by the interaction between the ionized and neutral molecules. The model is able to explain the experimentally obtained rotation of the molecules after the excitation of the system. Furthermore, we showed that the emergence of the negative charge in the substrate does not play a role in the rotation of the molecules.

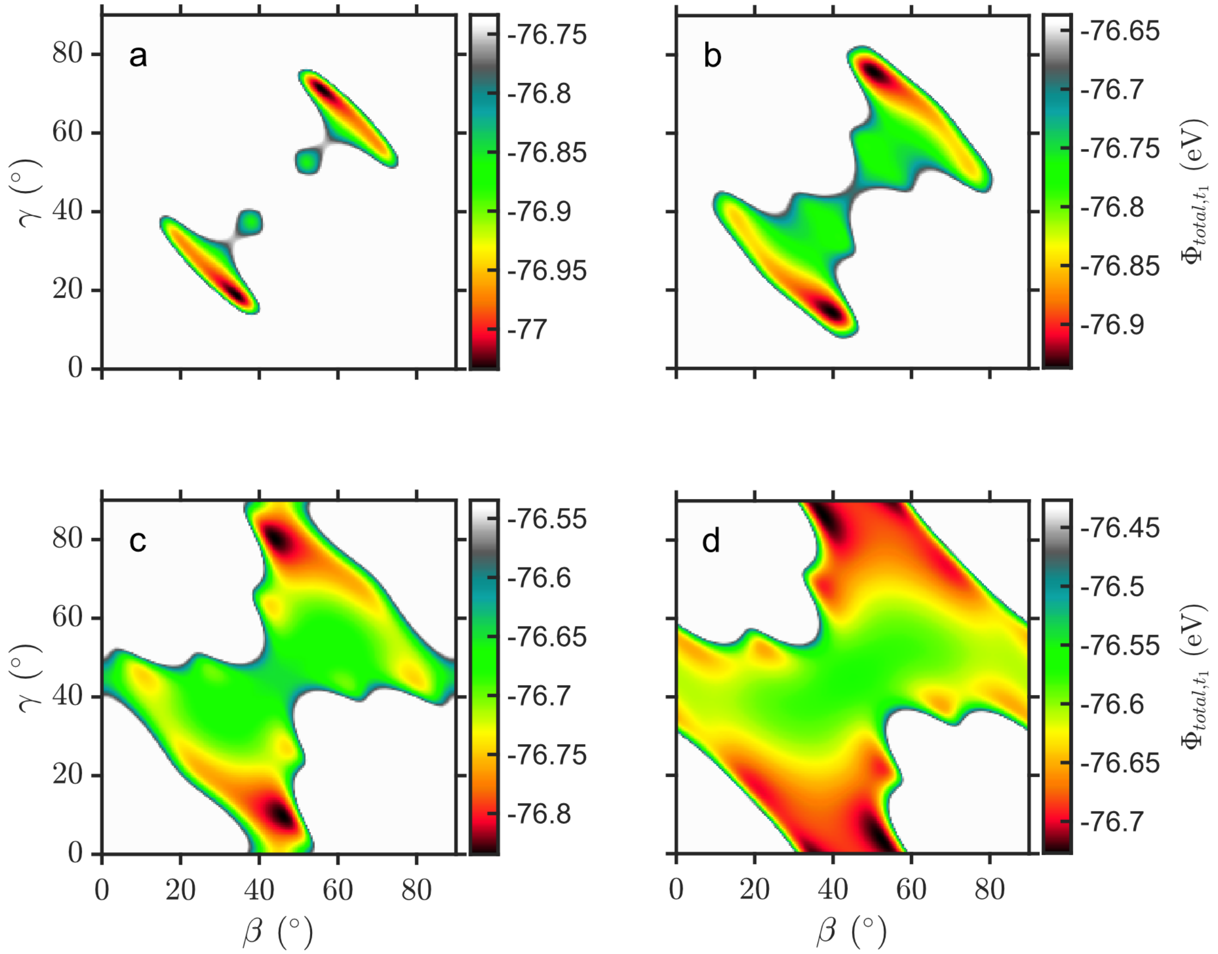

Supplementary Figure 25: Pair-potential Φ_{total,t_1} as a function of the orientation angles β and γ calculated for the excited structure taking the influence of the negative charge of the substrate into account. The pair-potential is shown for (a) $d = 14.0$ Å, (b) $d = 14.5$ Å, (c) $d = 15.0$ Å and (d) $d = 15.5$ Å, where d is the distance between next-neighbor molecules.

Supplementary references

1. Emmerich, S. *et al.* Ultrafast charge-transfer exciton dynamics in C60 thin films. *The Journal of Physical Chemistry C* **124**, 23579–23587 (2020). URL https://doi.org/10.1021/acs.jpcc.0c08011. PMID: 33193941, https://doi.org/10.1021/acs.jpcc.0c08011.

2. Curcio, D. *et al.* Ultrafast electronic linewidth broadening in the C \$1s\$ core level of graphene. *Physical Review B* **104**, L161104 (2021). URL https://link.aps.org/doi/10.1103/PhysRevB.104.L161104. Publisher: American Physical Society.

3. Stepanow, S. *et al.* Giant spin and orbital moment anisotropies of a cu-phthalocyanine monolayer. *Phys. Rev. B* **82**, 014405 (2010). URL https://link.aps.org/doi/10.1103/PhysRevB.82.014405.

4. Gordan, O., Friedrich, M. & Zahn, D. The anisotropic dielectric function for copper phthalocyanine thin films. *Organic Electronics* **5**, 291–297 (2004). URL https://www.sciencedirect.com/science/article/pii/S1566119904000795.

5. Porer, M. *et al.* Non-thermal separation of electronic and structural orders in a persisting charge density wave. *Nature Materials* **13**, 857–861 (2014). URL https://doi.org/10.1038/nmat4042.

6. Rösner, M., Şaşıoğlu, E., Friedrich, C., Blügel, S. & Wehling, T. O. Wannier function approach to realistic coulomb interactions in layered materials and heterostructures. *Phys. Rev. B* **92**, 085102 (2015). URL https://link.aps.org/doi/10.1103/PhysRevB.92.085102.

7. Andersen, K., Latini, S. & Thygesen, K. S. Dielectric genome of van der waals heterostructures. *Nano Letters* **15**, 4616–4621 (2015). URL https://doi.org/10.1021/acs.nanolett.5b01251. https://doi.org/10.1021/acs.nanolett.5b01251.

8. Kresse, G. & Hafner, J. Ab initio molecular dynamics for liquid metals. *Phys. Rev. B* **47**, 558–561 (1993). URL https://link.aps.org/doi/10.1103/PhysRevB.47.558.

9. Kresse, G. & Joubert, D. From ultrasoft pseudopotentials to the projector augmented-wave method. *Phys. Rev. B* **59**, 1758–1775 (1999). URL `https://link.aps.org/doi/10.1103/PhysRevB.59.1758`.

10. Lüftner, D. *et al.* Understanding the photoemission distribution of strongly interacting two-dimensional overlayers. *Phys. Rev. B* **96**, 125402 (2017). URL `https://link.aps.org/doi/10.1103/PhysRevB.96.125402`.

11. Perdew, J. P., Burke, K. & Ernzerhof, M. Generalized gradient approximation made simple. *Phys. Rev. Lett.* **77**, 3865–3868 (1996). URL `https://link.aps.org/doi/10.1103/PhysRevLett.77.3865`.

12. Singh, B., Hsu, C.-H., Tsai, W.-F., Pereira, V. M. & Lin, H. Stable charge density wave phase in a $1T$-$TiSe_2$ monolayer. *Phys. Rev. B* **95**, 245136 (2017). URL `https://link.aps.org/doi/10.1103/PhysRevB.95.245136`.

13. Bianco, R., Calandra, M. & Mauri, F. Electronic and vibrational properties of tise_2 in the charge-density-wave phase from first principles. *Phys. Rev. B* **92**, 094107 (2015). URL `https://link.aps.org/doi/10.1103/PhysRevB.92.094107`.

14. Grimme, S. Semiempirical gga-type density functional constructed with a long-range dispersion correction. *Journal of Computational Chemistry* **27**, 1787–1799 (2006). URL `https://onlinelibrary.wiley.com/doi/abs/10.1002/jcc.20495`. `https://onlinelibrary.wiley.com/doi/pdf/10.1002/jcc.20495`.

15. Popescu, V. & Zunger, A. Extracting $\boldsymbol{E}$ versus $\boldsymbol{k}$ effective band structure from supercell calculations on alloys and impurities. *Phys. Rev. B* **85**, 085201 (2012). URL `https://link.aps.org/doi/10.1103/PhysRevB.85.085201`.

16. Feibelman, P. J. & Eastman, D. E. Photoemission spectroscopy—correspondence between quantum theory and experimental phenomenology. *Phys. Rev. B* **10**, 4932–4947 (1974). URL `https://link.aps.org/doi/10.1103/PhysRevB.10.4932`.

17. Puschnig, P. *et al.* Reconstruction of molecular orbital densities from photoemission data. *Science (New York, N.Y.)* **326**, 702–706 (2009).

18. Niki, K., Shimokawa, H. & Nakato, A. Establishment of a calculation method for investigating surface characteristics using light. *e-J. Surf. Sci. Nanotech.* **22**, 334–341 (2024).

19. Frisch, M. J. *et al.* Gaussian 16 Revision C.01 (2016). Gaussian Inc. Wallingford CT.

20. Malmqvist, P. Å., Rendell, A. & Roos, B. O. The restricted active space self-consistent-field method, implemented with a split graph unitary group approach. *Journal of Physical Chemistry* **94**, 5477–5482 (1990).

21. Aquilante, F. *et al.* Molcas 8: New capabilities for multiconfigurational quantum chemical calculations across the periodic table. *Journal of Computational Chemistry* **37**, 506–541 (2016). URL https://onlinelibrary.wiley.com/doi/abs/10.1002/jcc.24221. https://onlinelibrary.wiley.com/doi/pdf/10.1002/jcc.24221.

22. Pierloot, K., Dumez, B., Widmark, P.-O. & Roos, B. O. Density matrix averaged atomic natural orbital (ano) basis sets for correlated molecular wave functions. *Theoretica chimica acta* **90**, 87–114 (1995).

23. Evangelista, F. *et al.* Electronic structure of copper phthalocyanine: An experimental and theoretical study of occupied and unoccupied levels. *The Journal of Chemical Physics* **126**, 124709 (2007). URL https://aip.scitation.org/doi/full/10.1063/1.2712435. Publisher: American Institute of Physics.

24. de Oteyza, D. G. *et al.* Copper-phthalocyanine based metal–organic interfaces: The effect of fluorination, the substrate, and its symmetry. *The Journal of Chemical Physics* **133**, 214703 (2010). URL https://doi.org/10.1063/1.3509394. https://doi.org/10.1063/1.3509394.

25. Nardi, M. V. *et al.* Electronic properties of CuPc and H2Pc: an experimental and theoretical study. *Phys. Chem. Chem. Phys.* **15**, 12864–12881 (2013). URL http://dx.doi.org/10.1039/C3CP51224J.

26. Puschnig, P., Koller, G., Draxl, C. & Ramsey, M. G. *Small Organic Molecules on Surfaces: Fundamentals and Applications*, 3–23 (Springer Berlin Heidelberg, Berlin, Heidelberg, 2013). URL http://dx.doi.org/10.1007/978-3-642-33848-9_1.

27. Puschnig, P. & Lüftner, D. Simulation of angle-resolved photoemission spectra by approximating the final state by a plane wave: From graphene to polycyclic aromatic hydrocarbon molecules. *Journal of Electron Spectroscopy and Related Phenomena* **200**, 193 – 208 (2015). URL http://www.sciencedirect.com/science/article/pii/S0368204815001309.

28. Popova-Gorelova, D., Küpper, J. & Santra, R. Imaging electron dynamics with time- and angle-resolved photoelectron spectroscopy. *Phys. Rev. A* **94**, 013412 (2016). URL https://link.aps.org/doi/10.1103/PhysRevA.94.013412.

29. Day, P., Wang, Z. & Pachter, R. Calculation of the structure and absorption spectra of phthalocyanines in the gas-phase and in solution. *Journal of Molecular Structure: THEOCHEM* **455**, 33–50 (1998). URL https://www.sciencedirect.com/science/article/pii/S0166128098002383.

30. Mastryukov, V., yu Ruan, C., Fink, M., Wang, Z. & Pachter, R. The molecular structure of copper- and nickel-phthalocyanine as determined by gas-phase electron diffraction and ab initio/dft computations. *Journal of Molecular Structure* **556**, 225–237 (2000). URL https://www.sciencedirect.com/science/article/pii/S0022286000006360.

31. Karlström, G. *et al.* Molcas: a program package for computational chemistry. *Computational Materials Science* **28**, 222–239 (2003).

32. Davidson, E. R., Ortiz, J. V. & Staroverov, V. N. Complete-active-space extended koopmans theorem method. *The Journal of Chemical Physics* **155**, 051102 (2021). URL https://doi.org/10.1063/5.0058080. https://doi.org/10.1063/5.0058080.

33. Kröger, I. *et al.* Modeling intermolecular interactions of physisorbed organic molecules using pair potential calculations. *The Journal of Chemical Physics* **135**, 234703 (2011). URL https://doi.org/10.1063/1.3665923. https://doi.org/10.1063/1.3665923.

34. Kleimann, C., Stadtmüller, B., Schröder, S. & Kumpf, C. Electrostatic interaction and commensurate registry at the heteromolecular F16CuPc–CuPc interface. *The Journal of Physical Chemistry C* **118**, 1652–1660 (2014). URL https://doi.org/10.1021/jp411289j. https://doi.org/10.1021/jp411289j.

35. Stadtmüller, B., Henneke, C., Soubatch, S., Tautz, F. S. & Kumpf, C. Tailoring metal–organic hybrid interfaces: heteromolecular structures with varying stoichiometry on ag(111). *New Journal of Physics* **17**, 023046 (2015). URL `https://doi.org/10.1088/1367-2630/17/2/023046`.

36. Kröger, I., Stadtmüller, B. & Kumpf, C. Submonolayer and multilayer growth of titaniumoxide-phthalocyanine on Ag(111). *New Journal of Physics* **18**, 113022 (2016). URL `https://doi.org/10.1088/1367-2630/18/11/113022`.

37. Felter, J., Franke, M., Wolters, J., Henneke, C. & Kumpf, C. Two-dimensional growth of dendritic islands of NTCDA on Cu(001) studied in real time. *Nanoscale* **11**, 1798–1812 (2019). URL `http://dx.doi.org/10.1039/C8NR08943D`.
