# Supplementary figures and images for: Femtosecond concerted rotation of molecules on a 2D material interface
Source: Nat Commun. 2026 Feb 27;17:2110. doi: 10.1038/s41467-026-69801-6 (PMC12954116; doi:10.1038/s41467-026-69801-6)

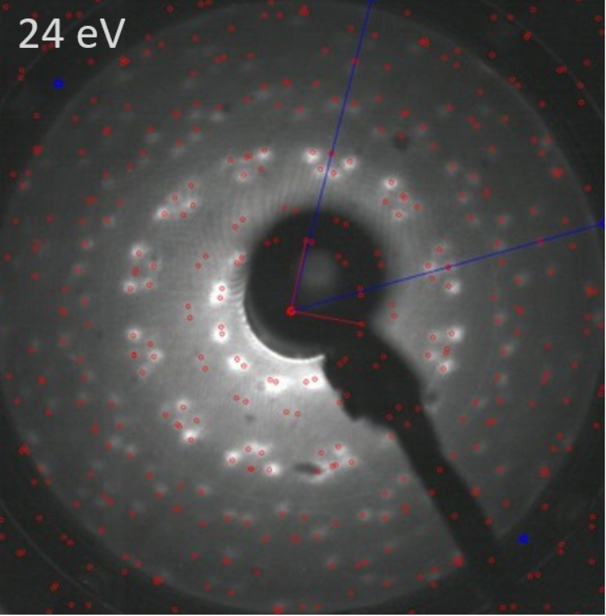

Supplement: Supplementary file 4 — Source data 2 [file 41467_2026_69801_MOESM4_ESM.zip › figS1.PNG]
